# Effect of Intrinsic Noise on the Phenotype of Cell Populations Featuring Solution Multiplicity: An Artificial *lac* Operon Network Paradigm

**DOI:** 10.1371/journal.pone.0132946

**Published:** 2015-07-17

**Authors:** Ioannis G. Aviziotis, Michail E. Kavousanakis, Andreas G. Boudouvis

**Affiliations:** School of Chemical Engineering, National Technical University of Athens, Athens, Greece; Tata Institute of Fundamental Research, INDIA

## Abstract

Heterogeneity in cell populations originates from two fundamentally different sources: the uneven distribution of intracellular content during cell division, and the stochastic fluctuations of regulatory molecules existing in small amounts. Discrete stochastic models can incorporate both sources of cell heterogeneity with sufficient accuracy in the description of an isogenic cell population; however, they lack efficiency when a systems level analysis is required, due to substantial computational requirements. In this work, we study the effect of cell heterogeneity in the behaviour of isogenic cell populations carrying the genetic network of *lac* operon, which exhibits solution multiplicity over a wide range of extracellular conditions. For such systems, the strategy of performing solely direct temporal solutions is a prohibitive task, since a large ensemble of initial states needs to be tested in order to drive the system—through long time simulations—to possible co-existing steady state solutions. We implement a multiscale computational framework, the so-called “equation-free” methodology, which enables the performance of numerical tasks, such as the computation of coarse steady state solutions and coarse bifurcation analysis. Dynamically stable and unstable solutions are computed and the effect of intrinsic noise on the range of bistability is efficiently investigated. The results are compared with the homogeneous model, which neglects all sources of heterogeneity, with the deterministic cell population balance model, as well as with a stochastic model neglecting the heterogeneity originating from intrinsic noise effects. We show that when the effect of intrinsic source of heterogeneity is intensified, the bistability range shifts towards higher extracellular inducer concentration values.

## Introduction

The phenotype of a cellular population is not exclusively the result of single-cell level complex chemical networks; cells interact with each other leading to phenotypic variations amongst the individual members of isogenic populations, a phenomenon commonly known as cellular heterogeneity. The literature reporting cellular heterogeneity is large and here we cite some representative examples, e.g., the variations of phage burst size [1], the transcriptional states heterogeneity in sporulating cultures of *Bacillus subtilis* [2], and the lysogenic states of phage-infected bacteria [3, 4]. The effect of heterogeneity has been studied in transcriptomics [5, 6], metabolomics [7], pathogens [8–12], as well as in mitochondrial activity [13–15]. It is also noteworthy to report that the design of contemporary biomedical therapies and of synthetic circuits with robust performance incorporates the effects of heterogeneity [16–18].

For an isogenic cell population residing in a uniform extracellular environment, there exist two fundamentally different sources of heterogeneity [19]: The first one originates from unequal partitioning of the mother intracellular content to its offsprings during division [20, 21]. The unevenly distributed regulatory molecules lead to different phenotypes, and the phenomenon is repeated due to the operation of the cell cycle. This type of heterogeneity is called *extrinsic* [19]. The regulatory molecules, which control the network of intracellular reactions and determine the cells phenotype exist in small amounts [22–24], and even small fluctuations can lead to an uncontrolled-uncertain outcome (phenotype). Thus, cells with *approximately* the same amount of regulatory molecules can feature utterly different phenotypic behaviour; this type of heterogeneity is called *intrinsic* [19].

Several models simulating heterogeneous populations have been developed in order to elucidate the effect of the different sources of heterogeneity. Shah et al. [25] were the first to model the stochastic behaviour of cell populations by developing a Monte Carlo algorithm for the dynamics of the cell mass distribution. Hatzis et al. [26] extended this algorithm to describe the dynamics of a growing population of phagotrophic protozoa. However, these models are computationally expensive due to the exponentially growing number of simulated cells of the population. To overcome the extensive requirements in CPU time, Constant-Number Monte Carlo (CNMC) algorithms are used [27, 28] simulating a constant number of cells that are assumed to be a representative sample of the studied population. More recent studies include the work of Shu et al. [29] in which the population balance models incorporate extrinsic heterogeneity and intracellular stochastic processes through $\text{It}\hat{o}$ stochastic differential equations; a chemical master equation for the population level, which models uncertainty of intracellular reactions, DNA duplication and content partitioning has been presented in [30–32]. Zechner et al. [33] used low-order moments through the moment closure approach to approximate intrinsic and extrinsic distributions; Toni and Tidor [17] employed van Kampen’s Ω-expansion for the approximation of intrinsic stochastic dynamics and incorporated extrinsic heterogeneity through variability of kinetic parameters and initial conditions. Finally, we report agent-based modelling approaches of cell, which have been presented in [34–37].

In this work, we apply a CNMC algorithm developed by Mantzaris [27] modelling the dynamics of an isogenic population. The algorithm takes into account the random nature of cell division, and unequal partitioning of intracellular content at cell division modelling extrinsic heterogeneity. In this model, interactions between individual cells are not taken into consideration. In addition, a Langevin approximation [19] of the reaction dynamics at the single-cell level is used to incorporate the effect of intrinsic heterogeneity. In our case study, all cells carry the *lac* operon genetic network [38, 39]; it is an artificial genetic network with a positive feedback architecture, featuring solution multiplicity within a range of extracellular inducer (IPTG, TMG or lactose) concentration values at the single-cell level. Bistability is also present at the population level, however the range of solution multiplicity is significantly altered. This has been demonstrated in [40, 41] by solving deterministic cell population balance models, which incorporate the effect of extrinsic heterogeneity. In order to quantify the effect of intrinsic heterogeneity, we need to apply stochastic modelling (here the CNMC algorithm), since deterministic models disregard the discrete nature of cell populations and cannot incorporate the intrinsic source of heterogeneity.

The disadvantage of stochastic modelling is its inefficiency to perform a systems level analysis of a population’s long time behaviour for a wide range of parameter values, by executing exclusively temporal simulations. In a recent publication [21], we demonstrated the application of a multiscale framework, which enables the application of well-established numerical algorithms utilizing short bursts of dynamic stochastic simulations. This framework is known as “equation-free” method [42–44]; it wraps around fine scale models interchanging information between microscopic (single-cell) and macroscopic (population) level, in order to perform numerical tasks including the computation of steady-state solutions, stability, and bifurcation analysis. The interchange of information between the different levels of description is feasible by constructing a discrete time-mapping, the *coarse time-stepper*, which reports the evolution of macroscopic quantities of interest at discrete time instances.

Here, we apply the equation-free method and wrap it around the CNMC model [27], which describes the dynamics of an isogenic cell population. When each individual of the population carries the *lac* operon genetic network, the existence of a range of extracellular inducer concentration values (IPTG) is expected, within which the population can feature utterly different phenotypic behaviour, i.e., high or low expression levels of the *lacY* gene can be both observed for the same parameter value. We apply bifurcation analysis, by means of the pseudo arc-length parameter continuation technique [45], in order to accurately determine the limits of the solution multiplicity region, which is then compared with the results obtained from the deterministic cell population balance models. This comparison, which quantifies the effect of intrinsic heterogeneity on the phenotype of a cell population, reveals some interesting findings. In particular, when the intrinsic heterogeneity effect is strengthened, the bistability interval is located at higher extracellular inducer IPTG concentration values. This bistability interval can be even expunged when we consider high asymmetry in the partitioning mechanism and sharper rates for the cells division. The accurate determination of the bistability interval is important for the understanding of possible phenotypic switching when the cell population operates at the proximity of the limits of this interval.

The paper is organized as follows: In the next section we present a simplified single-cell reaction rate expression, which describes the dynamics of the *lac* operon genetic network. In order to incorporate intrinsic noise effects, the reaction rate is augmented with a gaussian noise term (Langevin approach). We then briefly describe the homogeneous model, which neglects all sources of heterogeneity, the deterministic cell population balance model, which incorporates extrinsic heterogeneity, and finally the stochastic CNMC algorithm, which takes into account both extrinsic and intrinsic source of heterogeneity. Upon description of the stochastic CNMC model, we present the multiscale equation-free methodology, which enables the performance of coarse steady-state, bifurcation and stability analysis. In the Results section, we present steady state solutions of cell populations as a function of the IPTG concentration for different levels of intrinsic and extrinsic heterogeneity. Finally, we outline the main findings of this work and propose potent future research directions.

## Methods

### Single-Cell *Lac* Operon Dynamics

In this work, we study the dynamics of an isogenic population carrying the *lac* operon genetic network. It consists of a promoter (*lacP*), an operator (*lacO*) and three genes (*lacZ*, *lacY*, *lacA*) which encode the necessary proteins for the metabolism of lactose. The three *lac* operon genes are inhibited by a *lacI* repressor. The *lacI* repressor binds to the operator site, *lacO*, and prevents binding of the RNA polymerase inhibiting the transcription of the three genes’ DNA to the corresponding mRNA.

In the presence of lactose or its analogues, TMG or IPTG, the inducer is transported into the cell, binds to the repressor *lacI* through a bimolecular reaction and the operator *lacO* becomes free of *lacI*, hence initiating the transcription. Upon expression of *lacY*, the transport of the inducer is facilitated resulting in further expression of the three *lac* operon genes. Thus, the expression of *lacY* gene promotes its further expression, and the network functions as an autocatalytic system or a positive feedback loop [21, 40, 41].

A simplified description of the reaction steps has been described in [2, 40, 46] as follows:

$$\begin{array}{c} O_{0}+R\overset{k_{r}}{\underset{k_{-r}}{⇌}}O_{1} \end{array}$$ (1a)

$$\begin{array}{c} 2I+R\overset{k_{r2}}{\underset{k_{-r2}}{⇌}}I_{2}R \end{array}$$ (1b)

$$\begin{array}{c} I_{ex}\overset{k_{t}}{\underset{k_{t}}{⇌}}I \end{array}$$ (1c)

$$\begin{array}{c} O_{0}\overset{k_{Y}}{⟶}Y \end{array}$$ (1d)

$$\begin{array}{c} Y+I_{ex}\overset{k_{p}}{\underset{k_{-p}}{⇌}}YI_{ex}\overset{k_{ft}}{⟶}Y+I \end{array}$$ (1e)

$$\begin{array}{c} Y\overset{\lambda_{Y}}{⟶}• \end{array}$$ (1f)

$$\begin{array}{c} I\overset{\lambda_{I}}{⟶}•. \end{array}$$ (1g)

*O*_0_ and *O*_1_ denote the free and occupied operator sites. The repressor *R* *binds to and unbinds from the occupied and the free operator sites* at a rate proportional to *k*_*r*_ and *k*_−*r*_, respectively. The extracellular IPTG inducer, *I*_*ex*_, is transported through the cell membrane at a rate proportional to *k*_*t*_. Binding of the intracellular IPTG, *I*, to the repressor molecules occurs at rate proportional to *k*_*r*2_, and the rate constant of unbinding is *k*_−*r*2_. We also consider the rate of expression of the *lac* operon genes to produce *lac* permease, *Y*, to be proportional to the amount of free operator sites *O*_0_; *Lac* permease facilitates the transport of IPTG, playing the role of the enzyme and *I*_*ex*_ playing the role of the substrate in a scheme following the Michaelis-Menten kinetics. Finally, we assume that *Y* and *I* degrade following first-order kinetics at a rate constant *λ*_*Y*_ and *λ*_*I*_, respectively. Values of the kinetic constants are given in Table 1.

**Table 1:** Parameters of the *lac* operon reaction model.

Symbol	Value	Description
*V*_*E*.*coli*_	8 × 10^−16^ L	*E. coli* volume [47]
*O*_*T*_	2.08 nM	Operator Content (1 molecule) [48]
*k*_*Y*_	0.5 min^−1^	*lac*Y transcription rate constant [49]
*k*_*r*2_	3 × 10^−7^ nM^−2^min^−1^	Association rate constant of IPTG repression [48]
*k*_−*r*2_	3 × 10^3^ min^−1^	Dissociation rate constant of IPTG repression [48]
*k*_*r*_	960 nM^−1^min^−1^	Association rate constant of *O*_0_ repression [50]
*k*_−*r*_	60 min^−1^	Dissociation rate constant of *O*_0_ repression [50]
*k*_*p*_	1.32 nM^−1^min^−1^	LacY-IPTG association rate constant [2]
*k*_−*p*_	6 × 10^5^ min^−1^	LacY-IPTG dissociation rate constant [2]
*k*_*ft*_	6 × 10^4^ min^−1^	IPTG facilitated transport constant [2]
*λ*_*Y*_	0.0025 min^−1^	LacY degradation constant [40]
*λ*_*I*_	0.0025 min^−1^	IPTG degradation constant [40]

By assuming that the total number of operator sites and repressor molecules remain constant, i.e., *O*_*T*_ = *O*_0_ + *O*_1_, and *R*_*T*_ = *R* + *I*_2_ *R*, one can deduce a deterministic model describing the single-cell *lac* operon dynamics:

$$\begin{array}{cc} & \frac{d[Y]}{dt}=k_{Y}\left[O_{0}\right]+\left(k_{ft}+k_{-p}\right)\left[YI_{ex}\right]-k_{p}\left[Y\right]\left[I_{ex}\right]-\lambda_{Y}\left[Y\right] \end{array}$$ (2a)

$$\begin{array}{cc} & \frac{d[YI_{ex}]}{dt}=k_{p}\left[Y\right]\left[I_{ex}\right]-\left(k_{ft}+k_{-p}\right)\left[YI_{ex}\right] \end{array}$$ (2b)

$$\begin{array}{cc} & \frac{d[I]}{dt}=k_{-r2}\left(R_{T}-R\right)-k_{r2}{\left[I\right]}^{2}\left[R\right]+k_{t}\left[I_{ex}\right]+k_{ft}\left[YI_{ex}\right]-\lambda_{I}\left[I\right] \end{array}$$ (2c)

$$\begin{array}{cc} & \frac{d[R]}{dt}=k_{-r}\left(O_{T}-O_{0}\right)-k_{r}\left[O_{0}\right]\left[R\right]-k_{r2}{\left[I\right]}^{2}\left[R\right]+k_{-r2}\left(R_{T}-R\right) \end{array}$$ (2d)

$$\begin{array}{cc} & \frac{d[O_{0}]}{dt}=-k_{Y}O_{0}-k_{r}\left[O_{0}\right]\left[R\right]+k_{-r}\left(O_{T}-O_{0}\right). \end{array}$$ (2e)

The mathematical description of the *lac* operon dynamics can be further simplified by assuming that [2, 40]:

1. binding and unbinding of repressor to operator site *O*_0_ + *R* ⇌ *O*_1_ are in equilibrium, with equilibrium constant: *K*_1_ = [*O*_1_]/([*R*][*O*_0_])
2. the repressor inactivation reaction 2*I* + *R* ⇌ *I*_2_ *R* is in equilibrium with equilibrium constant: *K*_2_ = ([*I*_2_ *R*]/[*I*]^2^[*R*]) = (*R*_*T*_ − [*R*])/([*I*]^2^[*R*]).

Based on these assumptions, the *lac* operon dynamics can be described through the following set of ordinary differential equations for *Y*, and the intracellular IPTG:

$$\begin{array}{c} \frac{d[Y]}{dt}=k_{Y}\left[O_{T}\right]\frac{1+K_{1}{\left[I\right]}^{2}}{1+K_{1}{\left[I\right]}^{2}+K_{2}\left[R_{T}\right]}-\lambda_{Y}\left[Y\right] \end{array}$$ (3a)

$$\begin{array}{c} \frac{d[I]}{dt}=\frac{k_{ft}\left[I_{ex}\right]\left[Y\right]}{k_{-p}/k_{p}+\left[I_{ex}\right]}+k_{t}(\left[I_{ex}\right]-\left[I\right])-\lambda_{I}\left[I\right]. \end{array}$$ (3b)

By defining the following dimensionless quantities:

$$\begin{array}{cc} & \hat{t}=\kappa /\lambda_{Y}, \end{array}$$ (4a)

$$\begin{array}{cc} & \hat{y}=k_{Y}\left[O_{T}\right]\kappa /\lambda_{Y}, \end{array}$$ (4b)

$$\begin{array}{cc} & \hat{I}=\frac{\left[I_{ex}\right]}{\lambda_{Y}+k_{t}}(\frac{k_{ft}\hat{y}}{k_{-p}/k_{p}+\left[I_{ex}\right]}+k_{t}), \end{array}$$ (4c)

and setting the dimensionless time: $\tau =t/\hat{t}$, the dimensionless *lac* permease: $x=[Y]/\hat{y}$, and the dimensionless intracellular IPTG: $v=[I]/\hat{I}$, Eqs (3) become:

$$\begin{array}{c} \frac{dx}{d\tau }=\frac{\pi \rho +v^{2}}{\rho +v^{2}}-\kappa x \end{array}$$ (5a)

$$\begin{array}{c} \frac{\lambda_{Y}}{\lambda_{Y}+k_{t}}\frac{dv}{d\tau }=\sigma \left(x-1\right)+\kappa \left(1-v\right), \end{array}$$ (5b)

with *κ* a dimensionless degradation parameter and:

$$\begin{array}{cc} & \pi =\frac{1}{1+K_{2}\left[R_{T}\right]}, \end{array}$$ (6a)

$$\begin{array}{cc} & \rho =\frac{1+K_{2}\left[R_{T}\right]}{K_{1}{\hat{I}}^{2}}, \end{array}$$ (6b)

$$\begin{array}{cc} & \sigma =\kappa (\frac{k_{ft}\hat{y}}{k_{-p}/k_{p}+\left[I_{ex}\right]})/(k_{t}+\frac{k_{ft}\hat{y}}{k_{-p}/k_{p}+\left[I_{ex}\right]}). \end{array}$$ (6c)

By substituting with the values reported in Table 1: $\frac{\lambda_{Y}}{\lambda_{Y}+k_{t}}\approx 3\times 10^{-3}<<1$, which suggests that the dynamics of the dimensionless IPTG amount, *v*, are much faster compared to the dynamics of the dimensionless *lac*Y amount, *x*. Furthermore, *σ* ≈ *κ*, which yields: *x* ≈ *v*, and the *lac* operon dynamics can be described by the following equation for the dimensionless amount of *lac* permease:

$$\begin{array}{c} \frac{dx}{dt}\equiv R\left(x\right)=\frac{\pi \rho +x^{2}}{\rho +x^{2}}-\kappa x. \end{array}$$ (7)

**Fig 1:**
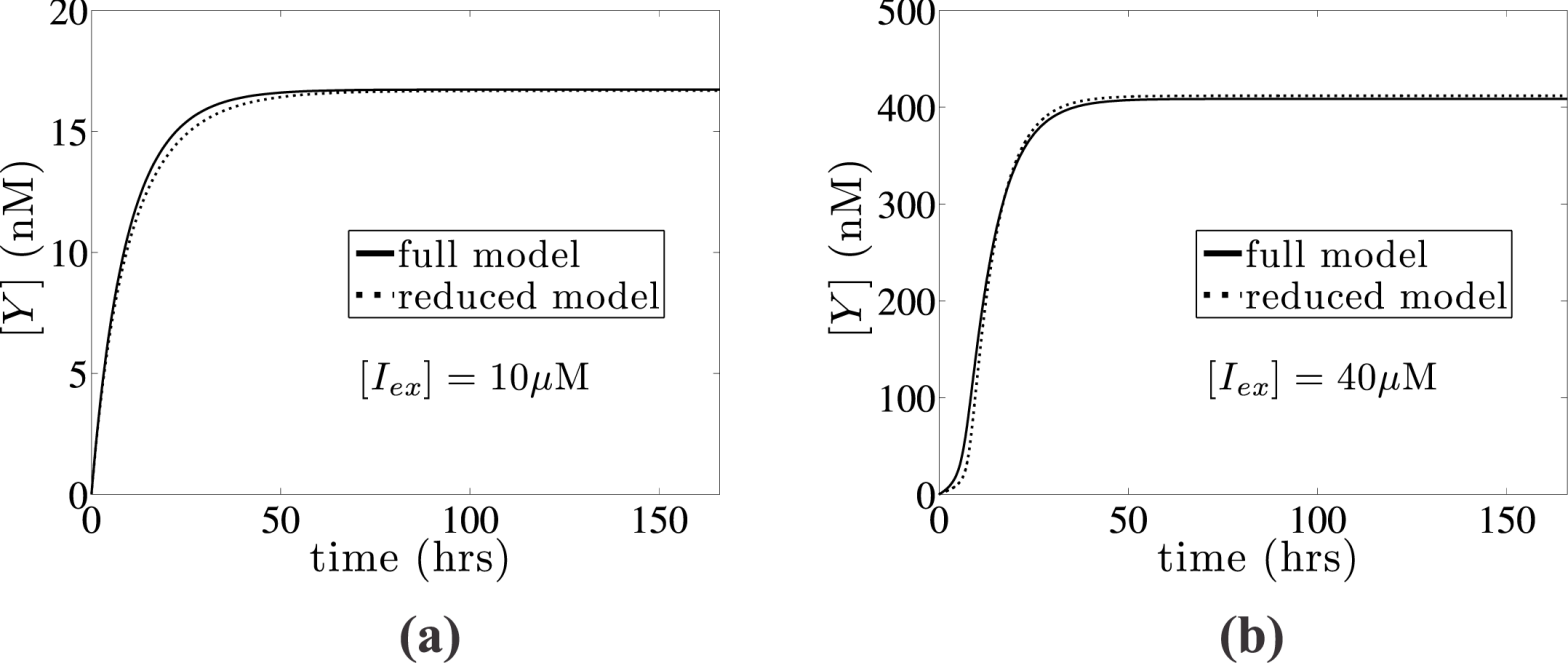
Reduction of deterministic model. Comparison between the full deterministic model (solid line) described by Eqs (2) with the reduced model (dashed line), which is described by Eq (7) for two different external IPTG concentrations leading to (a) an uninduced ([*I*_*ex*_] = 10*μ*M) and (b) an induced state ([*I*_*ex*_] = 40*μ*M). Kinetic constants are obtained from Table 1.

In order to numerically verify the validity of this reduction, we present in Fig 1 a comparison between the full deterministic model (Eqs (2)), and the reduced model (Eq (7)) for two different external IPTG concentration values leading to an uninduced (low *lac* permease amount) and an induced (high *lac* permease amount) state. In particular, we present the dynamics of the *lac* Y concentration for [*I*_*ex*_] = 10 and 40 *μ*M, showing good agreement between the full and reduced deterministic models. The *lac* permease concentration of roughly 400 nM at the fully induced state is in agreement with experimental data reported in [49].

### Langevin approximation of the single-cell reaction rate

The model described by Eq (7) is deterministic and it does not account for intrinsic noise effects, related with the small amount of regulatory molecules and slow operator fluctuations [19]. In order to take into account intrinsic noise effects, the reaction rate is re-formulated by adopting a Fokker-Planck approximation of the chemical master equation proposed in [19]. The approximation is based on the assumption that the operator fluctuations occur on a faster time scale compared to the production and degradation rate of the monomer. The monomer concentration which is denoted by *ρ*_*i*_((**x**), *t*) satisfies the equation:

$$\begin{array}{c} \vartheta_{t}\rho =L\left(x\right)\rho +K\rho , \end{array}$$ (8)

where **K** is the transition matrix containing the reaction rates for transitions between the operator’s chemical states and **L** is the diagonal matrix of the form:

$$\begin{array}{c} L_{ii}=-\sum_{j=1}^{q}\vartheta_{x_{j}}g_{ji}\left(x\right)+\frac{1}{2}\sum_{j=1}^{q}\vartheta_{x_{j}}^{2}h_{ji}\left(x\right). \end{array}$$ (9)

**g** is a matrix the *j* ^*th*^ column of which contains the net production rates of the *q* monomer species when the operator is in the *j* ^*th*^ chemical state. Likewise, **h** is the matrix the *j* ^*th*^ column of which contains the diffusion coefficients of the monomer species in the *j* ^*th*^ chemical state. Kepler and Elston [46] derived the following Fokker-Planck equation:

$$\begin{array}{c} \frac{\vartheta p(x,\tau )}{\vartheta \tau }=-\frac{\vartheta }{\vartheta x}\left[A\left(x\right)p\left(x,\tau \right)\right]+\frac{1}{2}\frac{\vartheta^{2}}{\vartheta x^{2}}\left[B\left(x\right)p\left(x,\tau \right)\right], \end{array}$$ (10)

where *p*(*x*, *τ*) is the probability density function of the random variable, *x* and:

$$\begin{array}{ccc} & & A(x)=\frac{k_{1}g_{0}+k_{0}g_{1}}{k_{0}+k_{1}}+\epsilon \frac{1}{k_{0}+k_{1}}\\ & & \left[k_{0}g_{0}\frac{\vartheta }{\vartheta x}(k_{0}\frac{g_{0}-g_{1}}{{\left(k_{0}+k_{1}\right)}^{2}})-k_{0}g_{1}\frac{\vartheta }{\vartheta x}(k_{1}\frac{g_{0}-g_{1}}{{\left(k_{0}+k_{1}\right)}^{2}})\right] \end{array}$$ (11)

$$\begin{array}{c} B\left(x\right)=2\epsilon \frac{k_{0}k_{1}{\left(g_{0}-g_{1}\right)}^{2}}{{\left(k_{0}+k_{1}\right)}^{3}}+\frac{h_{0}k_{1}+h_{1}k_{0}}{k_{0}+k_{1}}. \end{array}$$ (12)

Substitution of *g*_0_(*x*) = *π* − *κx*, *g*_1_(*x*) = 1 − *κx*, *h*_0_(*x*) = 1 + *κx*, *h*_1_(*x*) = *π* + *κx*, *k*_0_ = *x* ^2^, *k*_1_ = *ρ* and $\epsilon =\frac{1}{k}$, yields the following expressions for the drift and diffusion terms, *A*(*x*) and *B*(*x*), respectively:

$$\begin{array}{ccc} A(x) & = & \frac{\pi \rho +x^{2}}{\rho +x^{2}}-\kappa x-\\ & & \frac{2\rho x\left(\pi -1\right)[\left(\left(\pi -2\right)+\kappa x\right)x^{2}+\rho \left(\kappa x-\pi \right)]}{K{\left(\rho +x^{2}\right)}^{4}}, \end{array}$$ (13)

$$\begin{array}{ccc} B(x) & = & \frac{1}{y^{*}}[\frac{\rho \left(\pi +\kappa x\right)+x^{2}\left(1+\kappa x\right)}{\rho +x^{2}}]+\\ & & \frac{1}{K}[\frac{\rho x^{2}{\left(\pi -1\right)}^{2}}{{\left(\rho +x^{2}\right)}^{3}}], \end{array}$$ (14)

and the Langevin stochastic differential equation (SDE) for the reaction rate is given by:

$$\begin{array}{c} \frac{dx}{dt}\equiv R\left(x\right)=A\left(x\right)+\sqrt{B(x)}\xi \left(\tau \right), \end{array}$$ (15)

where *ξ*(*τ*) is a Gaussian white noise process.

**Fig 2:**
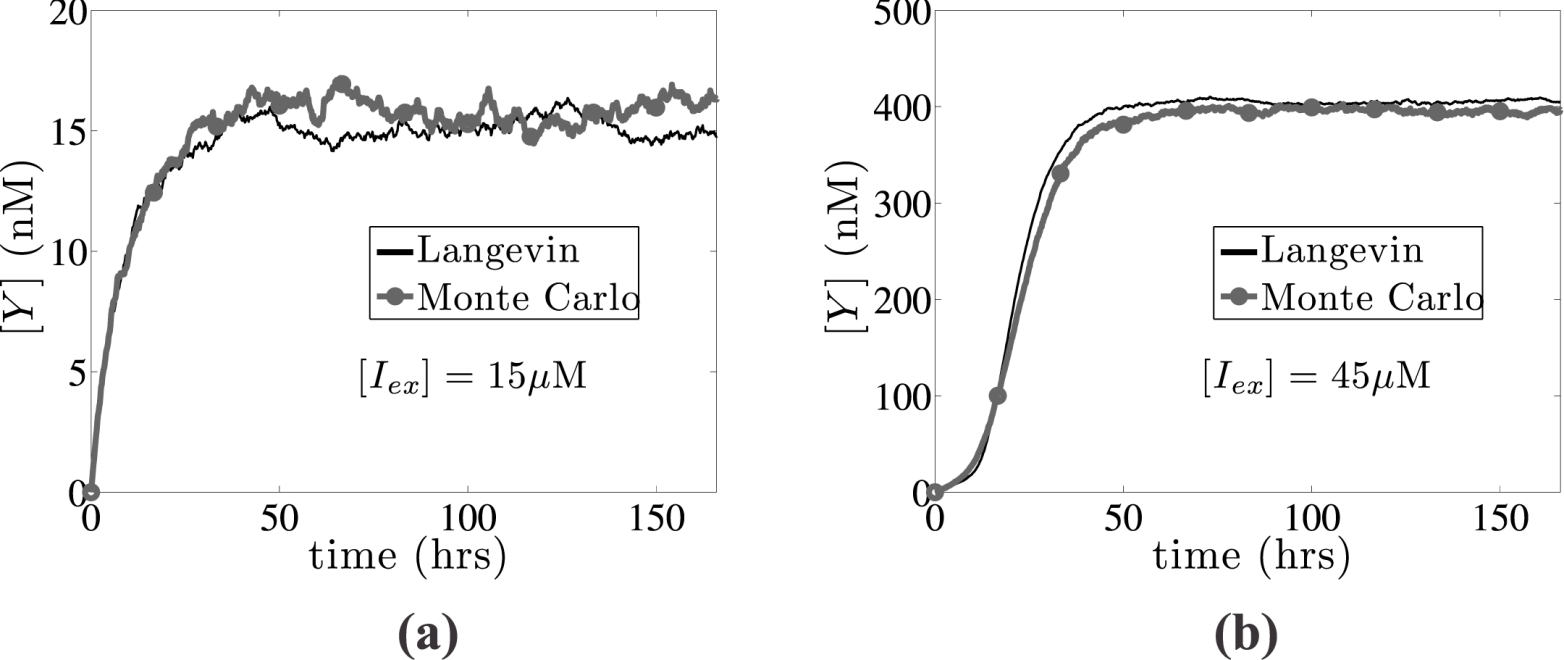
Langevin vs Monte Carlo single-cell *lac* operon simulations. Comparison between the Langevin approximation (black solid line) described by Eq (15) with Monte Carlo simulations (grey line with circles), using the Gillespie algorithm for the Master-equation of reactions Eqs (1) for two different external IPTG concentrations leading to (a) an uninduced ([*I*_*ex*_] = 15*μ*M) and (b) an induced state ([*I*_*ex*_] = 45*μ*M). 100 simulation copies are used to compute the average evolution.

The parameter *K* is a measure of the rate of the operator fluctuations and y* is the reference number of molecules, quantifying the two main sources of intrinsic heterogeneity, i.e., slow operator fluctuations and small numbers of molecules. The Langevin approximation is computationally advantageous over Monte Carlo simulations, by avoiding simulations of the full process, and sampling paths of the process (generated from Eq (15)) in shorter time [46]. We note that for very fast operator fluctuations (*K* → ∞) and/or large number of molecules (y* → ∞), the Langevin Eq (15) reduces to the deterministic expression of the reaction rate, Eq (7). Special treatment should be provided in cases, where the number of reference molecules is small enough (y* < 20), and the Langevin approach becomes unreliable to provide the probability of large deviation from the typical evolution (rare events) [51, 52]. However, in this work the results presented correspond to sufficiently large amount of *regulatory* molecules and sufficiently slow operator fluctuations, which can be accurately modelled by the efficient Langevin approximation. In Fig 2, we illustrate the accuracy of the Langevin approximation by comparing the dynamics of Eq (15) for y* = 20 and *K* = 200, with Monte Carlo simulations of the chemical Master-equation for the reaction system described by Eqs (1), using the direct Gillespie’s algorithm [53, 54]. In particular, we present the average evolution of 100 simulations of the single-cell *lac* operon dynamics for [*I*_*ex*_] = 15*μ*M, and [*I*_*ex*_] = 45*μ*M leading to an uninduced ([*Y*]_∞_ ≈ 15.5nM) and an induced ([*Y*]_∞_ ≈ 400nM) state, respectively.

The stochastic single-cell model (Eq (15)) is more realistic compared to the corresponding deterministic one (Eq (7)), since it can capture the intrinsic noise effects, however we are interested in studying the behaviour at the cell population level incorporating also the effect of heterogeneity.

### Deterministic Cell Population Balance Model

The extrinsic source of heterogeneity can be captured using a special class of models, which are commonly known as cell population balance models [19, 27, 41, 55]. In particular, we study the dynamics of the number density function *n*(*x*, *t*) corresponding to the number of cells per biovolume unit, which at time *t* have intracellular content between *x* and *x* + *dx* [55]:

$$\begin{array}{ccc} & & \frac{\partial n(x,t)}{\partial t}+\frac{\partial }{\partial x}\left[R\left(x\right)n\right]+\Gamma \left(x\right)n=\\ & & \;2\int_{x}^{x_{max}}\Gamma \left(x^{\prime }\right)P\left(x,x^{\prime }\right)n\left(x^{\prime },t\right)dx^{\prime }-n\int_{0}^{x_{max}}\Gamma \left(x\right)dx, \end{array}$$ (16)

where *R*(*x*) is the single-cell reaction rate (Eq (7)), which quantifies the rate of production or consumption of the intracellular content, *x*; Γ(*x*) is the division rate of each cell with content *x*. For the division rate we adopt the following normalized power law [56]:

$$\begin{array}{c} \Gamma \left(x\right)={\left(\frac{x}{\left\langle x\right\rangle }\right)}^{m}, \end{array}$$ (17)

where *m* regulates the sharpness of the division rate, and ⟨*x*⟩ is the average content of the population. *P*(*x*, *x*′) is a partition probability density function, which describes the mechanism of the distribution of a mother cell intracellular content among the two daughter cells. A simple formulation of the partition probability density function is:

$$\begin{array}{c} P\left(x,x^{\prime }\right)=\frac{1}{2f}\delta \left(fx^{\prime }-x\right)+\frac{1}{2(1-f)}\delta \left(\left(1-f\right)x^{\prime }-x\right), \end{array}$$ (18)

where *f* is the asymmetry parameter, and *δ* is the Dirac function. According to Eq (18), a mother cell with intracellular content *x* will provide a fraction *fx* to one of its offsprings, and the remaining (1 − *f*)*x* to the second one. The asymmetry parameter ranges within the interval [0,0.5], with lower values corresponding to asymmetric partitioning and *f* = 0.5 describing a symmetric partitioning mechanism. *R*(*x*), Γ(*x*), and *P*(*x*, *x*′) describe processes occurring at the single-cell level and are generally known as intrinsic physiological state functions (IPSF).

A simplification of the cell population balance model is the homogoneous model, which assumes that all cells behave like the average cell, with content ⟨*x*⟩_*h*_. The homogeneous model is derived by Eq (16), when the number density function is expressed as *n*(*x*, *t*) = *δ*(*x* − ⟨*x*⟩_*h*_) [2, 19, 41]. Then the dynamics of the homogenous population are given by the following differential equation:

$$\begin{array}{c} \frac{d{\left\langle x\right\rangle }_{h}}{dt}=R\left({\left\langle x\right\rangle }_{h}\right)-{\left\langle x\right\rangle }_{h}\overset{Eq.7}{=}\frac{\pi \rho +{\left\langle x\right\rangle }_{h}^{2}}{\rho +{\left\langle x\right\rangle }_{h}^{2}}-\kappa {\left\langle x\right\rangle }_{h}-{\left\langle x\right\rangle }_{h}. \end{array}$$ (19)

### The Stochastic CNMC Model

For a more realistic simulation of the dynamics of isogenic populations capturing all sources of heterogeneity, we implement the CNMC stochastic algorithm developed by Mantzaris [19], which is described below. We consider isogenic populations with cells carrying the same gene regulatory network, whose random state, *S*_*τ*_, at a dimensionless time instance, *τ*, is:

$$\begin{array}{c} S_{\tau }\equiv \left\{X_{i}\left(\tau \right)=x_{i},i=1,2,...,N\right\}. \end{array}$$ (20)

*X*_*i*_(*τ*) is the intracellular content of cell *i* and *N* is the constant number of cells considered for simulation.

*Time intervening two successive division events*

The time interval, *T*, intervening two successive division events is a random variable depending on *S*_*τ*_ with a cumulative distribution function given by the expression [27]:

$$\begin{array}{c} F_{T}\left(z|\tau \right)=1-exp[-\int_{0}^{z}\sum_{i=1}^{N}\Gamma (x_{i}(\tau +z^{\prime }))dz^{\prime }], \end{array}$$ (21)

where *z* denotes the set of time instances during which the next division event may occur for a given state, *S*_*τ*_. A random variable, *p*_1_, is generated from a uniform distribution [0, 1] and *T* is computed by solving the nonlinear equation:

$$\begin{array}{c} \frac{\int_{0}^{T}\sum_{i=1}^{N}\Gamma (x_{i}(\tau +z^{\prime }))dz^{\prime }}{\text{ln}[1-p_{1}]}+1=0. \end{array}$$ (22)

During the time interval, *T*, the intracellular content of each cell evolves according to the expression of *R*(*x*, *t*). Intrinsic noise is modelled using the Langevin SDE, Eq (15). The Gaussian white noise *ξ*(*τ*) is treated as a standard Brownian motion or a standard Wiener process and Eq (15) is solved using the $\text{It}\hat{o}$ interpretation of the stochastic integral [57, 58]. If the parameters of intrinsic noise are neglected, then the reaction rate of the intracellular content becomes deterministic (Eq (7)) and the stochastic algorithm accounts for effects of only extrinsic source of heterogeneity.

At the end of time interval, *T*, the cell undergoing division is selected from the conditional distribution function [27]:

$$\begin{array}{c} {\text{Pr}}_{k=j|S_{\tau +T}}=\frac{\Gamma (x_{j}\left(\tau +T\right))}{\sum_{i=1}^{N}\Gamma (x_{i}\left(\tau +T\right))}, \end{array}$$ (23)

where *k* is the index of the selected cell for division. Upon division of a cell, *k*, its content is distributed to its offsprings following a partition probability density function, *P*(*x*, *x*′). In this work, we select the simple discrete partitioning mechanism formulated by Eq (18), which generates a daughter cell with content *fx*_*k*_, and a second daughter cell with content (1 − *f*)*x*_*k*_. Finally, and in order to maintain the number of simulated cells constant, we randomly pick a cell which is replaced by the second offspring generated during division. The dimensionless time is updated, i.e., *τ* → *τ* + *T*, and the algorithm is repeated until a pre-specified time, *t*_*stop*_, is reached.

As stated above, the long time behaviour of an isogenic population carrying the *lac operon* genetic network is expected to feature bistable behaviour within a range of IPTG concentrations (∼ 1/*ρ*). Despite the fact that stochastic modelling provides a more realistic description of the population dynamics, it lacks efficiency when the systematic study of their long time behaviour is required, and especially in cases where multiple solutions can co-exist for the same IPTG concentration. In particular, exploring the solution space of (coarsely) time-invariant solutions over a wide range of parameter values, by executing solely temporal stochastic simulations is computationally demanding and renders the systematic study as a practically infeasible task. Furthermore, and since bistability regions are sought, when temporal simulations are performed at the vicinity of their intervals (critical turning points), stochastic noise can drive the population to unpredictable phenotypic switches.

### The equation-free methodology

When deterministic descriptions are available, the bistability limits can be tracked by means of bifurcation analysis applying well-established pseudo arc-length parameter continuation techniques as performed in [19, 41]. However, deterministic models are only simplified approximations failing into incorporating important information, e.g., originating from intrinsic noise effects.

**Fig 3:**
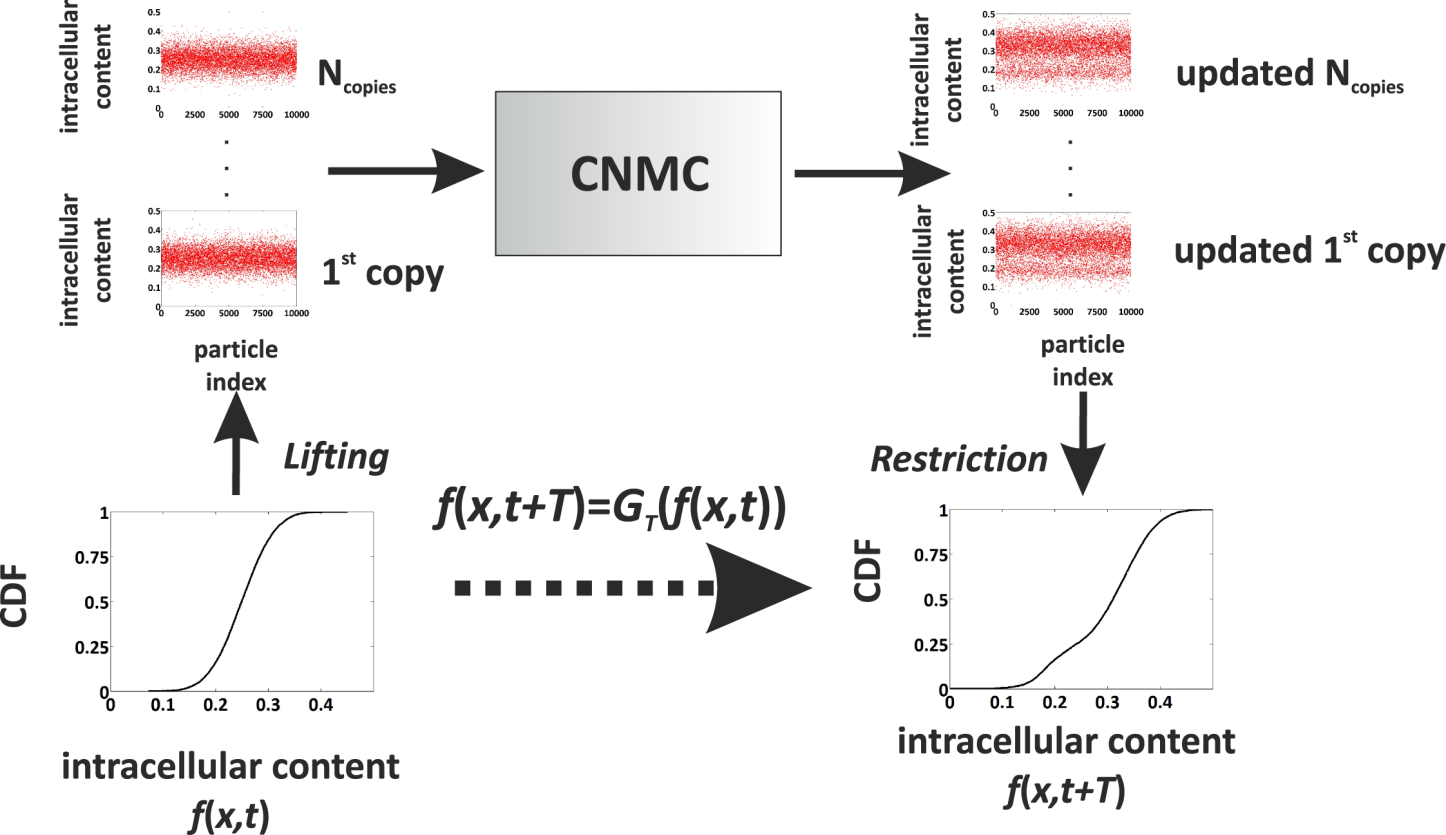
The coarse time-stepper. A schematic of the coarse time-stepper for the model of an isogenic cell population simulated by the CNMC algorithm.

More realistic descriptions are provided through stochastic simulations (e.g., the CNMC model), which however suffer from severe computational limitations; since we are interested in studying the long-time behaviour of cell populations over a wide range of parameter values, it is required to perform an extensive number of stochastic simulations, which renders the systematic study of the problem as a practically infeasible process. Alternatively, one can resort to multiscale computational techniques, such as the equation-free methodology, which utilises information originating from fine-scale (microscopic) level simulations, projects it to a coarse-macroscopic level, and enables the performance of numerical tasks, such as steady-state computations, stability and bifurcation analysis. The interchange of information between micro- and macro-scopic level is enabled through a computational structure, the *coarse time-stepper* [43, 44, 59, 60], which reports the evolution of macroscopic variables of interest at distinct time instances and is schematically illustrated in Fig 3; a macroscopic variable is a statistical measure of the microscopically simulated cell population, e.g., the Cumulative Distribution Function (CDF) of the intracellular content, which is denoted with *f*(*x*, *t*). Through the lifting step, we generate a number, N_copies_, of random states which are consistent with the studied coarse variable (CDF distribution). Each of these microscopic states are simulated with the CNMC model for a short time period, *T*, and the average CDF of the updated microscopic states is computed through the *restriction* step. In effect, the steps described above construct a discrete time-mapping:

$$\begin{array}{c} f\left(x,t+T\right)=G_{T}(f(x,T)) \end{array}$$ (24)

where the operator *G*_*T*_ is unknown. Below, we describe in detail the Restriction and Lifting procedures used in this study.

#### Restriction

If we denote with *x*_*i*_ the content of the *i* ^*th*^ cell, then the CDF is computed by sorting into ascending order the **x** = {*x*_*i*_}, *i* = 1, …, *N* vector, and plotting it against the vector **p** = {*p*_*i*_ = (*i* − 0.5)/*N*)}, *i* = 1, …, *N*. This constitutes a restriction of microscopic data, **U**, to the macroscopic variable, *f* through an operator, 𝓜, i.e., *f* = 𝓜**U**. For noise reduction purposes, many repetitions of the stochastic simulation are required and then the average restriction is computed.

#### Lifting

For the lifting step, we choose to work with the inverse CDF (ICDF), **x(p)**, which produces the intracellular content *x*_*i*_ = *x*(*p*_*i*_) of a given cell, *i*. Assuming, that the ICDF is smooth enough, and given that it has a finite support, *p* ∈ [0, 1], it can be approximated using a low-dimensional description, e.g., the first few of an appropriate sequence of orthogonal polynomials [60]. These polynomials are approximated by a series of *j*—degree orthogonal polynomials, *ϕ*_*j*_(*p*), which are monotone, non-decreasing functions and lie within the interval [0, 1]:

$$\begin{array}{c} x\left(p\right)\approx \sum_{j=0}^{q}\alpha_{j}\phi_{j}\left(p\right). \end{array}$$ (25)

The *ϕ*_*j*_ polynomials are represented by their values on the point set **p** and the first four basis functions (*q* = 3) are sufficient for the description of the corresponding fine scale state. In cases where the ICDF is not smooth enough, a larger number of basis functions are required. The analytical expressions of the basis functions up to 3^*rd*^ order degree are given below [21]:

$$\begin{array}{ccc} \phi_{0} & = & 1\\ \phi_{1} & = & 4.5953\tilde{p}-2.2977\\ \phi_{2} & = & 19.3299{\tilde{p}}^{2}-19.3299\tilde{p}+3.9171\\ \phi_{3} & = & 79.4133{\tilde{p}}^{3}-119.1200{\tilde{p}}^{2}+51.3112\tilde{p}-5.8023, \end{array}$$ (26)

where $\tilde{p}=0.5-\text{arcsin}(1-2p)/\pi$. Each of the polynomial basis functions is computed on the point set **p** constructing a (*q* + 1) × *N* matrix, Φ. The coefficients *α* = *α*_*i*_ are computed from:

$$\begin{array}{c} \alpha =\Phi x, \end{array}$$ (27)

and the microscopic state **x** can be approximated from:

$$\begin{array}{c} x\approx \Phi^{T}\alpha . \end{array}$$ (28)

Thus, the intracellular content of each cell of the population can be constructed by a small set of *α* values. The lifting procedure described above constructs a microscopic description **U** from a macroscopic variable *f* through an operator, *μ*, i.e., **U** = *μf*. It should be noted here that the choice of lifting and restriction operators needs to guarantee that lifting from coarse level to fine scale and then restricting to coarse level again has no effect, i.e., *μ*𝓜 ≈ 𝓘.

#### Healing Time

The naturally arisen question is whether the level of description using 4 basis functions is sufficient enough, and higher order approximation is required. To test this, we perform the following computational experiment: A CNMC simulation is interrupted at time *τ* = *τ*_*inter*_ and the microscopic state **x**_*original*_(*τ*_*inter*_) is obtained. Then, we perform the lifting step using 4 basis functions and construct a lifted microscopic state **x**_*lifted*_(*τ*_*inter*_). The next step is to perform two different CNMC simulations for a time horizon *T*_*hor*_ using as initial microscopic states: (a) **x**_*original*_(*τ*_*inter*_) and (b) the lifted **x**_*lift*_(*τ*_*inter*_). Finally, we compute the evolution of the *α* values computed using 5 = 4+1 basis functions for the two different simulations at distinct reporting time instances, and compare their relative error, (*α* ^*lift*^ − *α* ^*original*^)/*α* ^*original*^.

**Fig 4:**
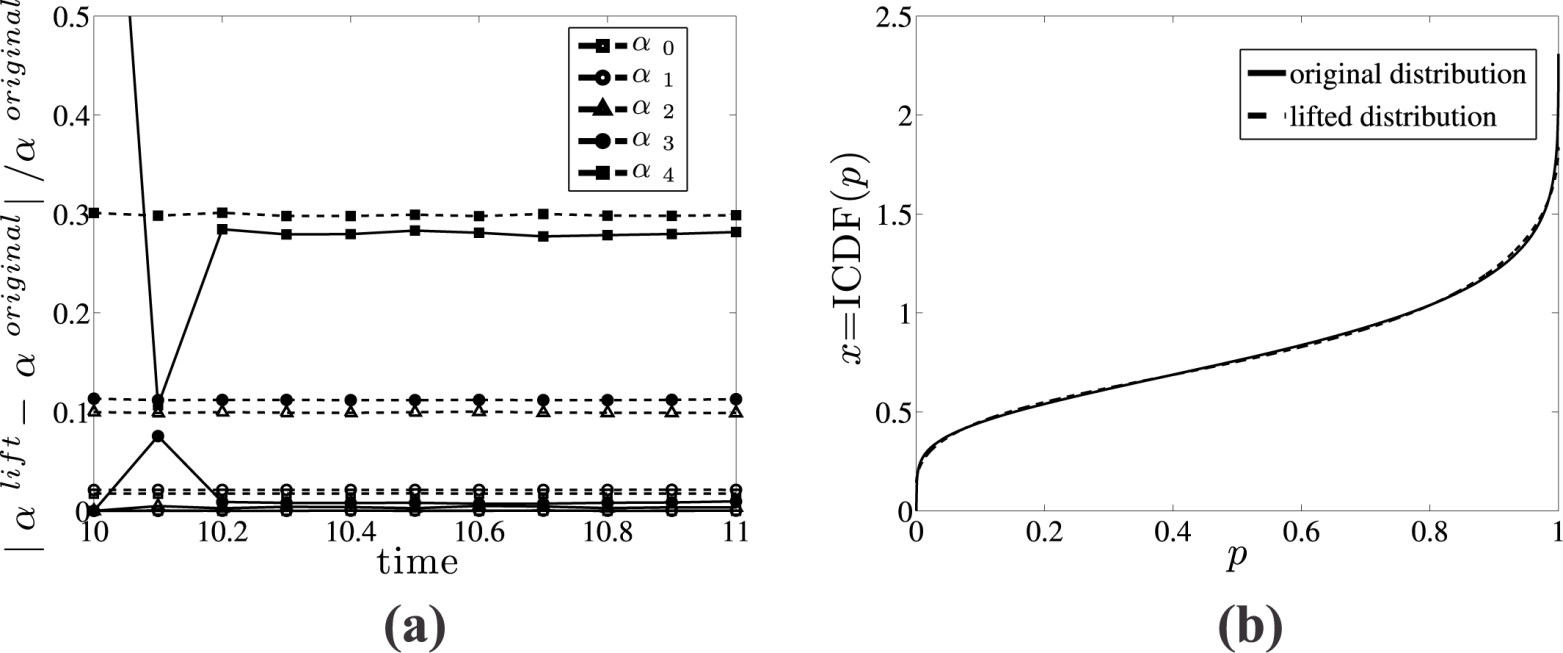
Computation of healing time. (a) Relative error between five “original” coefficients and coefficients computed after lifting. The *solid lines* correspond to relative errors, while *dashed lines* correspond to stochastic noise quantified by $2\sigma_{\alpha_{j}}/\alpha_{j}^{original}$; *σ*_*α*_*j*__ is the standard deviation of the noise of coefficients *α*_*j*_ computed using 50 direct CNMC copies. (b) The original (*solid line*) and the lifted (*dashed line*) ICDF distributions using 4 orthogonal basis functions at dimensionless time *τ* = 10.5.

In Fig 4(a) we show an example of this test computation, where a CNMC simulation is interrupted at dimensionless time *τ* = 10 to obtain the **x**_*original*_(10) and the lifted distribution **x**_*lifted*_(10) using four *α* coefficients (*q* = 3). Then the original and lifted distributions are simulated over a time interval *τ* ∈ [10, 11], and at distinct time instances with an interval of Δ*τ* = 0.1, we compute the five first *α* coefficients using a 4^*th*^ order approximation. As expected, the 5^*th*^ *α* coefficient of the lifted distribution is significantly different from the respective *α* coefficient of the original distribution; however, it takes only a small time interval of Δ*τ* = 0.1 to see that the 5^*th*^ *α* coefficient of both the original and lifted distributions have no difference (their relative difference drops below the stochastic noise computed from $2\sigma_{\alpha_{j}}/\alpha_{j}^{original}$, where *σ*_*α*_*j*__ is the standard deviation of the noise of coefficients calculated using a number (here 50) of direct CNMC copies [61]). The time required for higher order coefficients of lifted distributions to converge to the ones obtained from original distributions is referred to as *healing time* [42, 43], and it is a preparation time interval for the coarse-time stepper during which the dynamics of fast variables (higher order coefficients or moments of the distribution) equilibrate quickly and get slaved by the evolution of the lower order coefficients. The first four coefficients show no difference within the entire time interval *τ* ∈ [10, 11].

We also illustrate that using a low order approximation does not affect the shape of the distribution of cells as a function of their intracellular content. In particular, in Fig 4(b) we show the original and the lifted with up to 3^*rd*^ order polynomial approximation ICDF distributions. Clearly, lifting using a 3^*rd*^ order approximation (or 4 orthogonal basis functions) is accurate enough for a coarse-level description of the cell population.

#### Coarse steady state computations and parametric analysis

As reported above, the lifting and restriction steps constitute the coarse time-stepper, which maps the dynamics of the coarse variable *f* at discrete time instances according to the general expression of Eq (24), or if we study the coarse variable *α* according to the general expression:

$$\begin{array}{c} \alpha \left(t+T\right)={𝓖}_{T}\left(\alpha \left(t\right)\right) \end{array}$$ (29)

with the operator 𝓖_*τ*_ being unknown. This black-box simulator can be utilised for the performance of numerical tasks, such as steady state computations with the Newton-Raphson method. A steady-state solution, α* satisfies:

$$\begin{array}{c} \alpha^{*}-{𝓖}_{T}(\alpha^{*})=0. \end{array}$$ (30)

If we are interested in applying the Newton-Raphson method for the computation of the coarse steady-state solution α*, it is required to solve the following set of non-linear equations:

$$\begin{array}{c} R\equiv \alpha^{*}-{𝓖}_{T}\left(\alpha^{*}\right)=0. \end{array}$$ (31)

In order to solve the non-linear system of equations with α* being the unknowns we apply the Newton-Raphson method, which requires during each iteration the solution of the linearised system:

$$\begin{array}{c} \frac{\vartheta R}{\vartheta \alpha }\delta \alpha =[I-\frac{\vartheta {𝓖}_{T}}{\vartheta \alpha }]=-R \end{array}$$ (32)

Since the explicit expression of 𝓖_*T*_ is not available, its approximation is performed numerically using a simple forward finite difference scheme:

$$\begin{array}{c} {\left(\frac{\vartheta {𝓖}_{T}}{\vartheta \alpha }\right)}_{i,j}\approx \frac{{\left({𝓖}_{T}\left(\alpha_{j}+\epsilon \right)\right)}_{i}-{\left({𝓖}_{T}\left(\alpha_{j}\right)\right)}_{i}}{\epsilon }, \end{array}$$ (33)

where *ϵ* is a small perturbation number.

**Fig 5:**
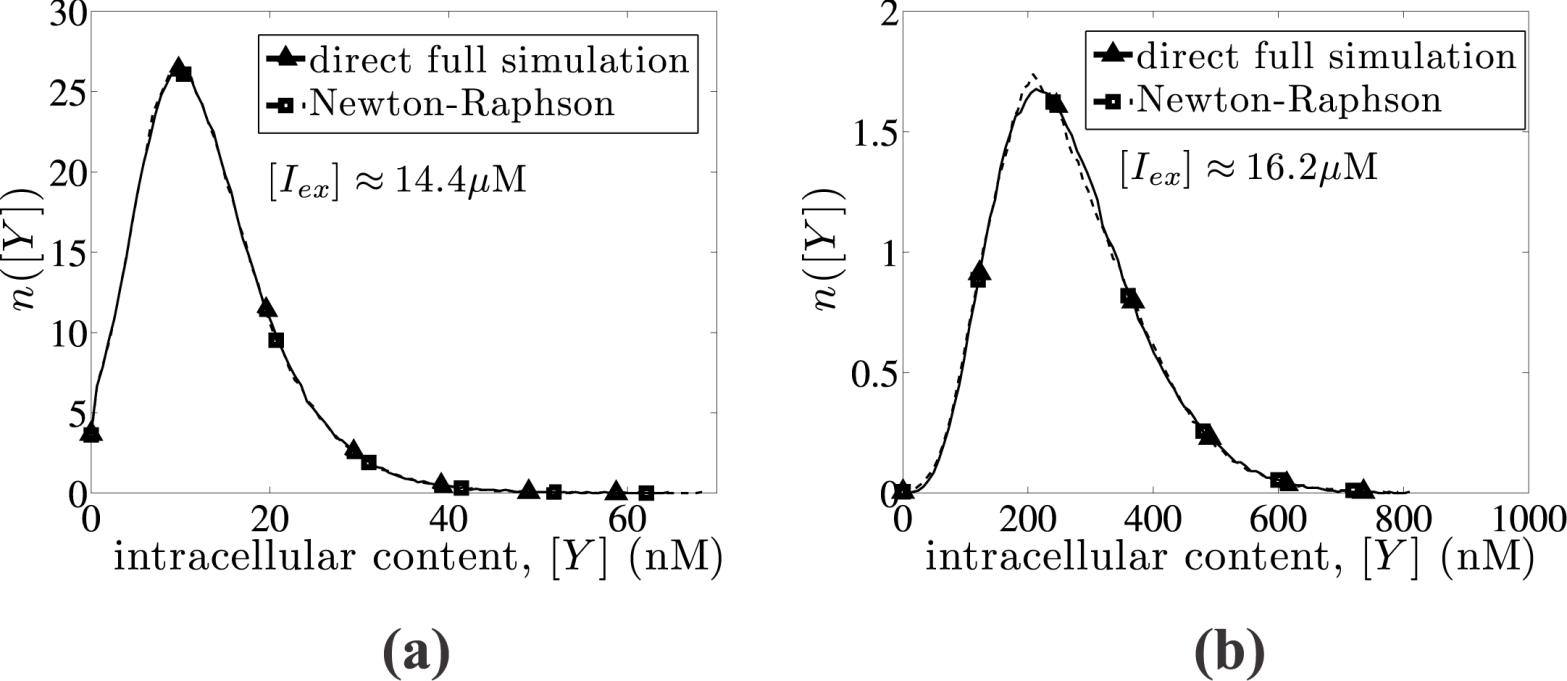
Comparison of full long temporal simulations with coarse steady state computations. Solid lines with open triangles correspond to number density functions, *n*([*Y*]), obtained from long temporal simulations, who have practically reached a steady state. Dashed lines with open rectangles correspond to number density functions obtained from Newton-Rapshon coarse steady state computations. Good agreement is observed for both (a) low level and (b) high level *lacY* expression levels. Parameter values: *K* = 500, y* = 50, *π* = 0.03, *m* = 2, *f* = 0.5, *N* = 10,000 cells. We use 50 copies of CNMC simulations for stochastic noise reduction purposes.

In Fig 5, we compare the number density function, *n*([*Y*] as a function of the intracellular content, [*Y*] as computed from (a) a direct temporal simulation of the full CNMC model performed until time, *t* ≈ 60 hrs, and (b) the equation-free based, Newton-Raphson computation described above. In particular, we compare the steady-state distributions for two cases of cell populations featuring low and high level *lac* permease concentration levels, showing very good agreement in both cases. It is evidently clear that the equation-free approach, even though uses a low-level description, it produces number cell density functions which compare remarkable well with the time consuming, full simulations of the CNMC model.

Using the same idea, one can perform bifurcation analysis by means of pseudo arc-length parameter continuation enabling the computation of the entire solution space, including stable and unstable steady state solutions. As a by-product of this process, the bistability range is accurately computed. Below, we present the results of this analysis quantifying the effect of intrinsic noise effect on heterogenous cell populations, by choosing as continuation parameter *the external IPTG concentration, [*I*_*ex*_]*.

## Results

### Effect of intrinsic noise

In order to decompose the effect of the different sources of heterogeneity, we perform the following steps. First, we solve the steady state solution of the homogeneous model (Eq (19)), which neglects all sources of heterogeneity and considers populations, where each individual cell carries the same intracellular amount. The extrinsic source of heterogeneity is then incorporated by solving the DCPB model (Eq (16)); for details of the numerical solution of the DCPB model, we refer the reader to Kavousanakis et al [41]. Finally, we employ the equation-free method to perform coarse bifurcation analysis of the stochastic model (CNMC) for different values of parameters *K* and y*, which quantify the effect of intrinsic noise.

**Fig 6:**
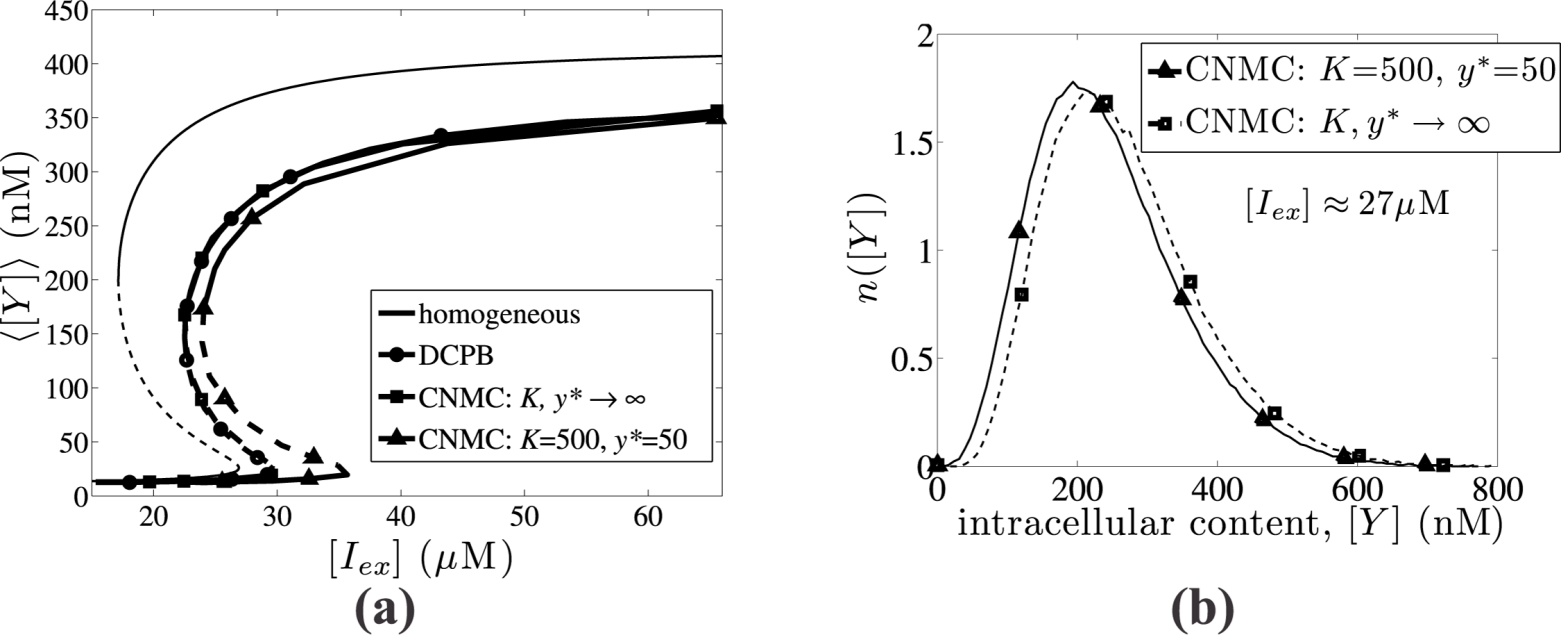
Effect of intrinsic heterogeneity. (a) Steady state average expression level, ⟨[*Y*]⟩, as a function of the external IPTG concentration, [*I*_*ex*_]. The *black lines (solid and dashed)* correspond to the homogeneous model, the *lines with open circles* to the DCPB model; the *lines with open squares* correspond to the CNMC model neglecting the intrinsic source of heterogeneity and the *lines with open triangles* to the CNMC model incorporating intrinsic noise effects. (b) Steady state solutions of the number density function, *n*([*Y*]), corresponding to the upper stable solution branches of CNMC simulations for [*I*_*ex*_] ≈ 27*μ*M, when intrinsic noise is incorporated (*K* = 500, y* = 50) and when neglected (*K*, y* → ∞).

In Fig 6(a), we present a bifurcation diagram showing the dependence of the average *lacY* expression of a population carrying the *lac operon* genetic network on the extracellular inducer concentration, [*I*_*ex*_] for four different cases: (a) the homogeneous model, (b) the DCPB model solved with the finite element method [41], (c) the CNMC model neglecting intrinsic noise effects (*K*, y* → ∞), and (d) the CNMC model incorporating the intrinsic source of heterogeneity. The single-cell reaction rate is given from Eq (7), with *π* = 0.03 and *κ* = 0.05. The partitioning mechanism is discrete (see Eq (18)) and symmetric (*f* = 0.5); the division rate is given from Eq (17) with *m* = 2. Finally, CNMC models simulate populations consisting of *N* = 10,000 cells; we use 50 copies of CNMC simulations for each parameter value for stochastic noise reduction purposes.

In all cases, one can observe an S-shaped bifurcation diagram with two stable steady state solution branches (solid lines) and a branch of unstable solutions (dashed lines). In Fig 6(b), we depict the average number density distribution of 50 copies of CNMC simulations *n*([*Y*]), for [*I*_*ex*_] ≈ 27*μ*M corresponding to the upper stable solution branch. The dashed line with open rectangles corresponds to the steady state distribution of cells as obtained from CNMC simulations, which neglect the effect of intrinsic noise (*K*, y* → ∞) and the solid line with open triangles corresponds to CNMC simulations with *K* = 500 and y* = 50, i.e., incorporating the effect of intrinsic noise. The average phenotype, ⟨[*Y*]⟩, is lower when the intrinsic source of heterogeneity is taken into account.

When the population resides in an environment, which is rich in the inducer IPTG, then the average expression level of *lacY* is high. By decreasing the external IPTG concentration, there exists a critical value (left turning point) which signals the abrupt transition towards low expression level of *lacY*. In a reverse experiment, where we start with low IPTG concentration values, the average expression of *lacY* is low, and by increasing IPTG there exists a critical value (right turning point) beyond which the average expression will jump towards higher values. The two turning points are distinct suggesting that transitions between populations featuring high and low *lacY* expression levels, through modification of the IPTG concentration are hysteretic.

The homogeneous model results show that the bistability region spans over a wide range of [*I*_*ex*_] values: [*I*_*ex*_] ∈ [17.1,26.9]*μ*M. Extrinsic heterogeneity shifts the bistability range towards higher [*I*_*ex*_] values (DCPB and CNMC with *K*, y* → ∞). Here, the results obtained from DCPB and CNMC show very good agreement, since the size of the cell population simulated by the CNMC model is large (10,000). The range of solution multiplicity is shifted further towards higher [*I*_*ex*_] values, by taking into account the effect of intrinsic noise (CNMC model with finite *K* and y* values). In particular, we report that the lower limit of the bistability region as computed from the CNMC model for *K* = 500 and y* = 50 is located at [*I*_*ex*_] = 24.1*μ*M compared to the value of [*I*_*ex*_] = 22.5*μ*M, which is computed when neglecting the intrinsic source of heterogeneity. In addition, the upper [*I*_*ex*_] limit of the bistability region is shifted towards higher values for the CNMC model with intrinsic noise ([*I*_*ex*_] = 35.7*μ*M) compared to [*I*_*ex*_] = 29.6*μ*M, for the CNMC model with *K*, y* → ∞. Thus, the combined effect of extrinsic and intrinsic heterogeneity shifts the bistability region towards higher IPTG concentration values. It should be noted here that similar findings have been presented in the experimental work of Maeda and Sano [62] reporting hysteric transitions within IPTG concentration values of 2.5 − 50*μ*M (for certain wild type promoters), as well as in Matsumoto et al. [63] reporting bistability in the range of 5–15*μ*M.

**Fig 7:**
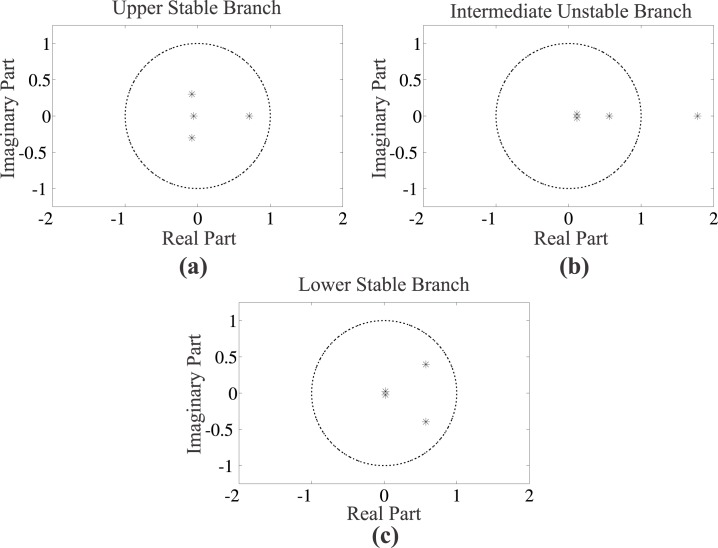
Coarse stability analysis. Eigenvalues of the $\frac{\vartheta {𝓖}_{\tau }(\alpha^{*})}{\vartheta \alpha }$ matrix corresponding to (a) the upper branch stable steady state, (b) the intermediate branch unstable steady state, and (c) the lower branch stable steady state solution, for [*I*_*ex*_] = 28.8*μ*M (*ρ* = 0.09). The loss of stability in (b) is marked by the eigenvalue crossing the unit circle (dashed line) in the complex plane. Parameter set values: *f* = 0.5, *m* = 2, *π* = 0.03, *κ* = 0.05, *K* = 500, *y* = 50, and *N* = 10,000 cells.

In order to characterise a steady state solution as stable or unstable, we perform coarse stability analysis by determining the eigenvalues of the $\frac{\vartheta {𝓖}_{\tau }(\alpha^{*})}{\vartheta \alpha }$ matrix (see Eq (32)). We present an indicative case for steady state solutions obtained from the model incorporating intrinsic heterogeneity (lines with open triangles in Fig 6(a)), and in particular for parameter value [*I*_*ex*_] = 28.8*μ*M. The spectra of eigenvalues presented in Fig 7(a)-7(c) correspond to the upper, intermediate, and lower branch, respectively. In Fig 7(a) and 7(c), the eigenvalues of the matrix, $\frac{\vartheta {𝓖}_{\tau }(\alpha^{*})}{\vartheta \alpha }$, lie within the limits of the complex plane unit circle, and the two solutions are dynamically stable. On the contrary, in Fig 7(b), one eigenvalue crosses the complex plane unit circle, and the corresponding steady state solution is characterised as dynamically unstable.

### Effect of operator fluctuations and reference number of molecules

**Fig 8:**
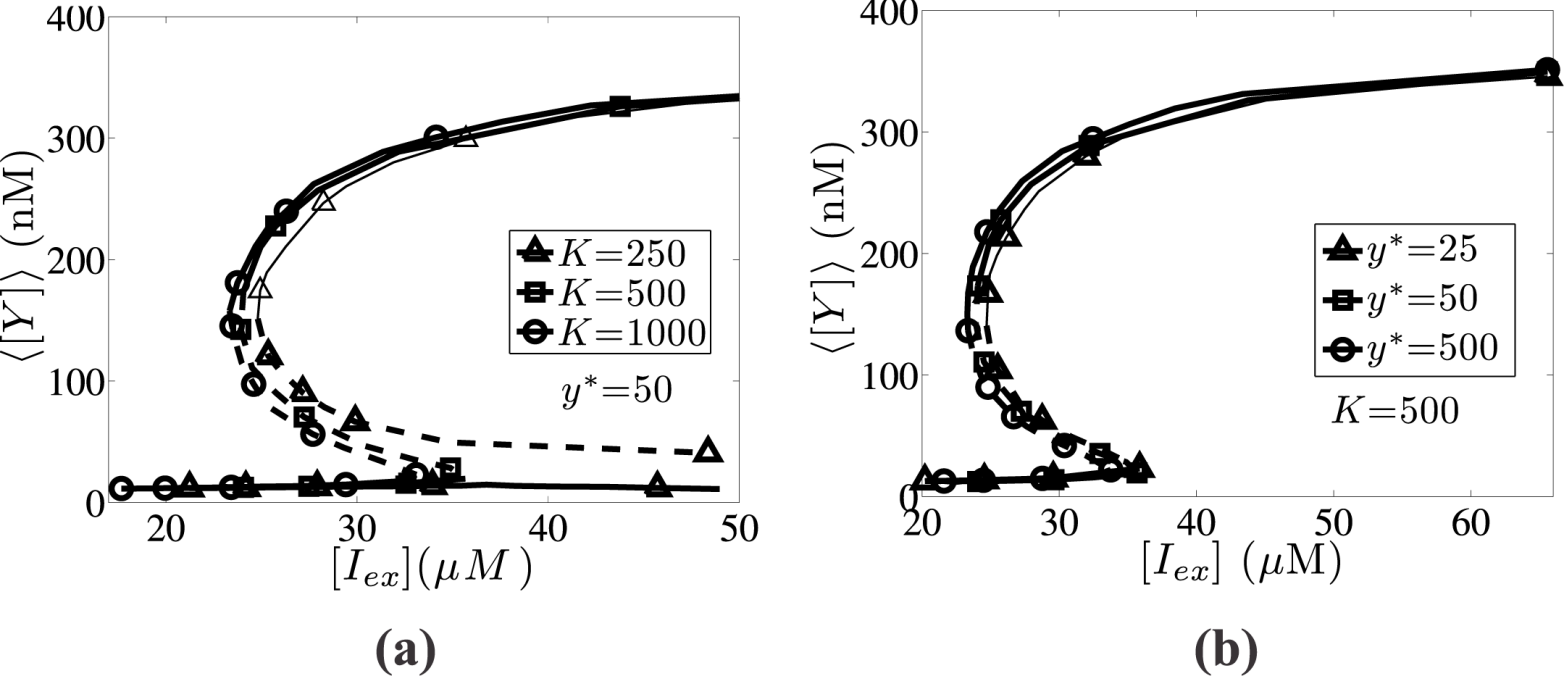
Effect of operator fluctuations, *K*, and reference number of molecules, y*. Effect of different sources of intrinsic heterogeneity on the average expression, ⟨[*Y*]⟩, as a function of the external IPTG concentration, [*I*_*ex*_]: (a) Effect of parameter *K*: *lines with open circles* correspond to *K* = 1000, *lines with open squares* correspond to *K* = 500 and *lines with open triangles* correspond to *K* = 250. (b) Effect of parameter y*: *lines with open circles* correspond to y* = 500, *lines with open squares* correspond to y* = 50 and *lines with open triangles* correspond to y* = 25. In both figures, solid and dashed lines denote stable and unstable steady state solutions, respectively. Parameter set values: *f* = 0.5, *m* = 2, *π* = 0.03 and *κ* = 0.05.

We now investigate the effect of the intrinsic heterogeneity “intensity”, which is quantified by the *K* and y* values. In particular, the effect of intrinsic noise is strengthened by lowering the operator fluctuations (*K*) and the reference number of regulatory molecules (y*). We demonstrate this effect in Fig 8, where the dependence of the steady state expression level of the average intracellular content, ⟨[*Y*]⟩, on the extracellular IPTG concentration, [*I*_*ex*_], is presented for different *K* and y* values. In Fig 8(a), we illustrate this effect by lowering the parameter *K* and keeping the parameter y* constant. By lowering the *K* value, the intrinsic noise effect is intensified and the bistability region is shifted towards higher, [*I*_*ex*_], values.

In particular, the bistability range for *K* = 1000 lies within the inerval [*I*_*ex*_] ∈ [23.3,33.1]*μ*M, whereas the corresponding interval for *K* = 250 starts from [*I*_*ex*_] = 24.8*μ*M and ranges up to unrealistically high (tending to infinity) values (the dimensionless *ρ* tends to 0). A similar trend is observed by lowering the y* parameter value with *K* fixed (see Fig 8(b)), however the degree of change is not as significant as in the variable, *K*, case. In this case, the bistability range for y* = 500 spans over the interval [*I*_*ex*_] ∈ [23.3,33.7]*μ*M, and lies within the interval [*I*_*ex*_] ∈ [24.7,35.7]*μ*M for y* = 25.

**Fig 9:**
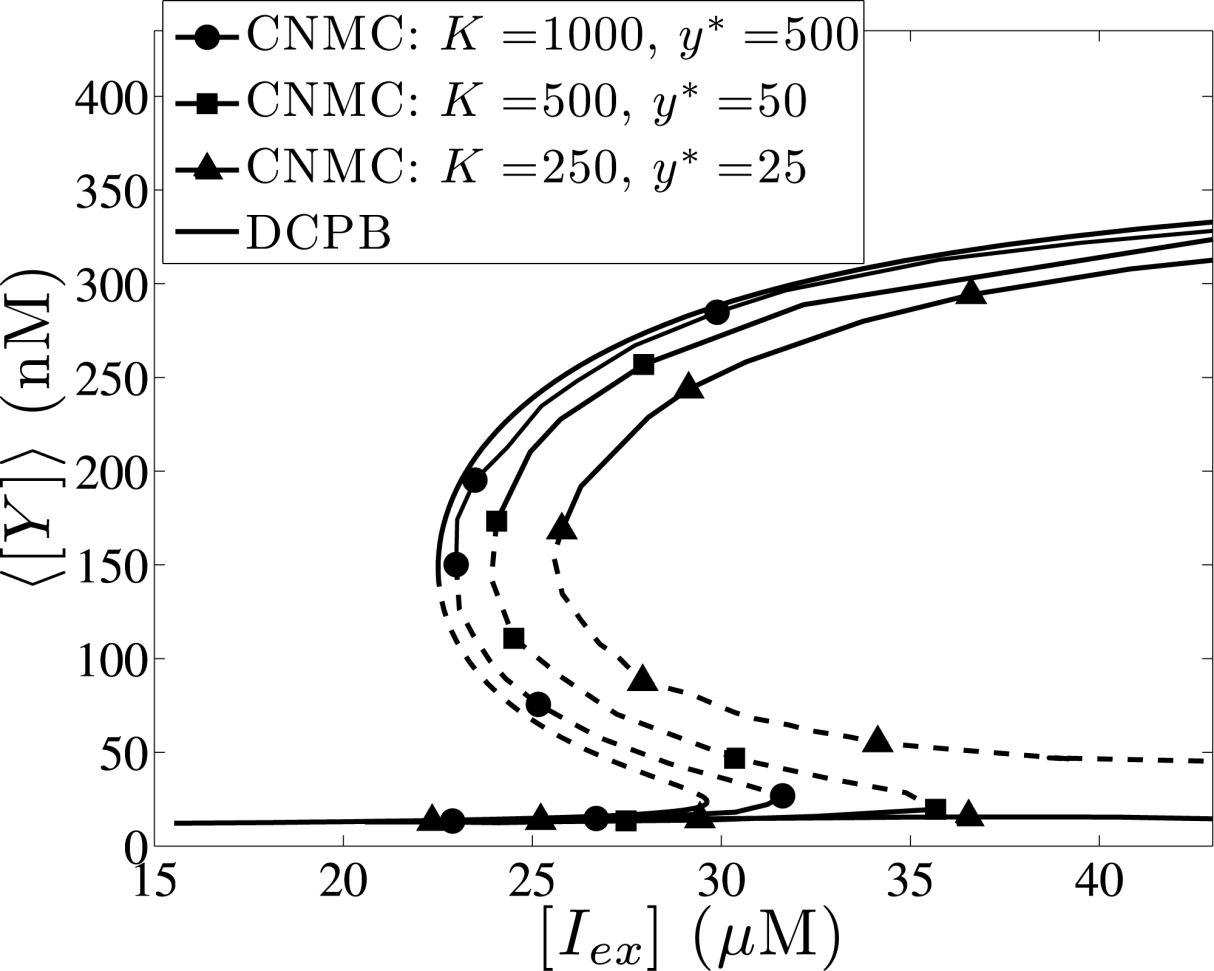
Effect of intrinsic noise intensity. Steady state average expression level, ⟨[*Y*]⟩, as a function of the external IPTG concentration, [*I*_*ex*_], for different *K* and y* values. The *lines with full circles* correspond to *K* = 1000 and y* = 500, the *lines with full squares* to *K* = 500 and y* = 50 and the *lines with full triangles* to *K* = 250 and y* = 25; the *black lines* correspond to the DCPB model. Stochastic simulations are performed with *N* = 10,000 cells (average of 50 copies for noise reduction). Parameter set values: *f* = 0.5, *m* = 2, *π* = 0.03 and *κ* = 0.05.

In the cases presented above, one of the two intrinsic noise parameters is kept constant and the intrinsic heterogeneity effect is strengthened by lowering the value of the other parameter. The two parameters of intrinsic source of heterogeneity act in a collaborative manner and strengthen further the effect of intrinsic noise by decreasing both *K* and y* values. In Fig 9, we compare the results obtained from the DCPB model with the CNMC model for: (a) *K* = 1000, y* = 500, (b) *K* = 500, y* = 50 and (c) *K* = 250, y* = 25. The CNMC model with the largest *K* and y* values resembles best the behaviour of the DCPB model, whereas low *K* and y* values shift the bistability limits towards higher [*I*_*ex*_] values. Interestingly enough, when the effect of intrinsic noise is sufficiently intensified, the right turning point reaches infinity (*ρ* → 0) and does not correspond to IPTG values with physical meaning suggesting that transitions between the high and low level expression states are non reversible: i.e., a high to low *lacY* expression level transition is feasible by decreasing the IPTG concentration; however the reverse transition becomes infeasible considering *K* = 250 and y* = 25, since the upper end of the bistability region is located at [*I*_*ex*_] → ∞ values.

### Effect of cell division sharpness and asymmetric partitioning

**Fig 10:**
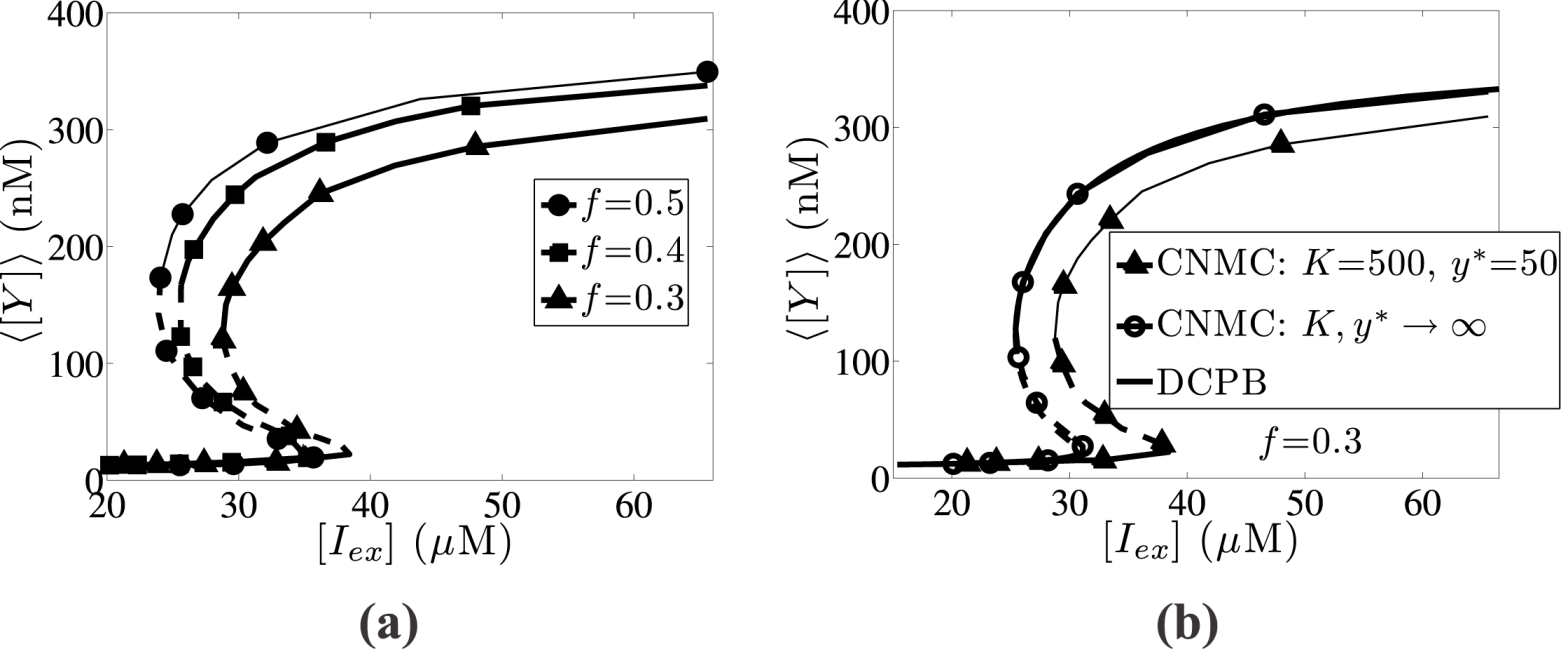
Effect of partitioning asymmetry parameter. Effect of the partitioning asymmetry parameter, *f*, in the average expression of *lacY* gene steady state (⟨[*Y*]⟩), as a function of the inverse IPTG concentration ([*I*_*ex*_]). (a) *Lines with full circles* correspond to symmetric partitioning (*f* = 0.5); *lines with full squares* correspond to *f* = 0.4 and *lines with full triangles* to *f* = 0.3. (b) Comparison between the CNMC model neglecting intrinsic source of heterogeneity with *f* = 0.3 (*lines with open circles*), the DCPB model (*black lines (solid and dashed)*) and the CNMC incorporating intrinsic noise effects (*lines with full triangles*). Parameter set values: *m* = 2, *π* = 0.03 and *κ* = 0.05. CNMC simulations are performed with *N* = 10,000 cells.

In addition to intrinsic noise parameters, we also examine the effect of cell division sharpness, quantified by parameter, *m*, and asymmetric partitioning, which is quantified by parameter, *f*. The effect of asymmetric partitioning is illustrated in Fig 10(a) for a population of *N* = 10,000 cells, showing the dependence of the average *lacY* content on the external IPTG concentration values for different *f* values. By increasing the asymmetry factor (i.e., lowering *f*), the bistability interval shrinks and shifts towards higher [*I*_*ex*_] values. In particular, the bistability region for symmetric partitioning (*f* = 0.5) lies within the interval [*I*_*ex*_] ∈ [13.5,20]*μ*M, whereas for *f* = 0.3 in the interval [*I*_*ex*_] ∈ [16.2,21.5]*μ*M.

The impact of asymmetric partitioning becomes more significant when combined with intrinsic heterogeneity as shown in Fig 10(b) for *f* = 0.3. In particular, the CNMC model neglecting intrinsic noise effects (*K*, y* → ∞) agrees well with the corresponding DCPB model, whereas the CNMC model with *K* = 500 and y* = 50 predicts a shifted towards higher [*I*_*ex*_] values bistability interval.

**Fig 11:**
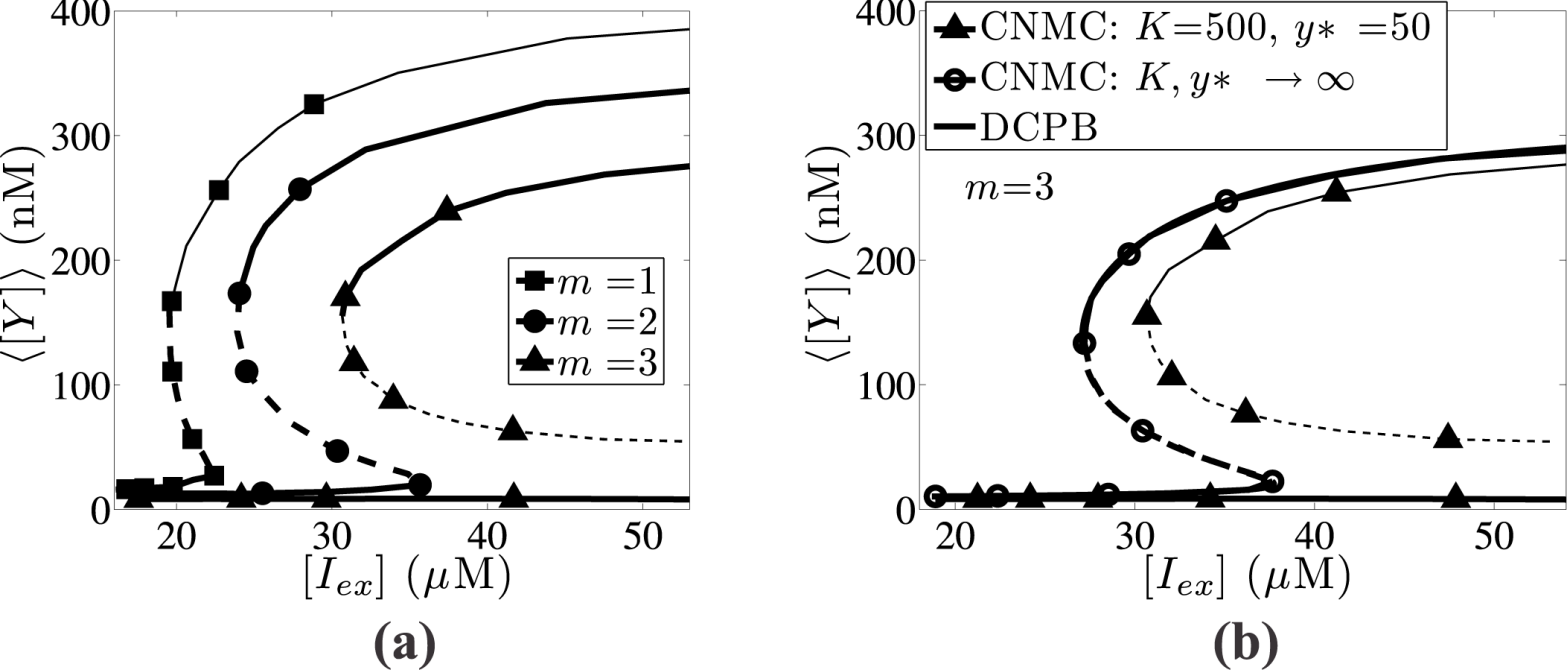
Effect of the sharpness division parameter. Effect of the sharpness division parameter, *m*, on the average expression of *lacY* gene steady state. (a) CNMC simulations of 10,000 cells with *K* = 500 and y* = 50. *Lines with full squares* correspond to *m* = 1, *lines with full circles* to *m* = 2 and *lines with full triangles* to the largest division rate, *m* = 3. (b) Comparison between the CNMC model with *m* = 3 (*lines with full triangles*) with the DCPB model (*black lines (solid and dashed)*) and the CNMC model neglecting intrinsic noise effects (*lines with open circles*). Parameter set values: *f* = 0.5, *π* = 0.03 and *κ* = 0.05.

Furthermore, the effect of division rate sharpness for a population of *N* = 10,000 is presented in Fig 11(a). Higher single-cell division rates (larger *m* values) shift its upper end towards higher [*I*_*ex*_] values. In particular, when *m* = 3 the upper end tends to infinity suggesting that transitions between high and low *lacY* expression levels are irreversible.

In Fig 11(b), we compare the CNMC model for *m* = 3 and *K* = 500, y* = 50 with the corresponding DCPB model and the stochastic CNMC model, which neglects intrinsic noise effects (*K*, y* → ∞). By incorporating the intrinsic source of heterogeneity, the effect of asymmetric partitioning becomes more intense leading to large discrepancies, compared to the ones between the DCPB model and the CNMC model, which neglects the intrinsic source of heterogeneity. In particular, the bistability regions for the CNMC with *K*, y* → ∞, and the DCPB model are [*I*_*ex*_] ∈ [27.3,37.8]*μ*M and [*I*_*ex*_] ∈ [27.1,37.8]*μ*M, respectively; the bistability interval for the CNMC model with *K* = 500 and y* = 50 spans over [*I*_*ex*_] ∈ [30.6, ∞]*μ*M.

**Fig 12:**
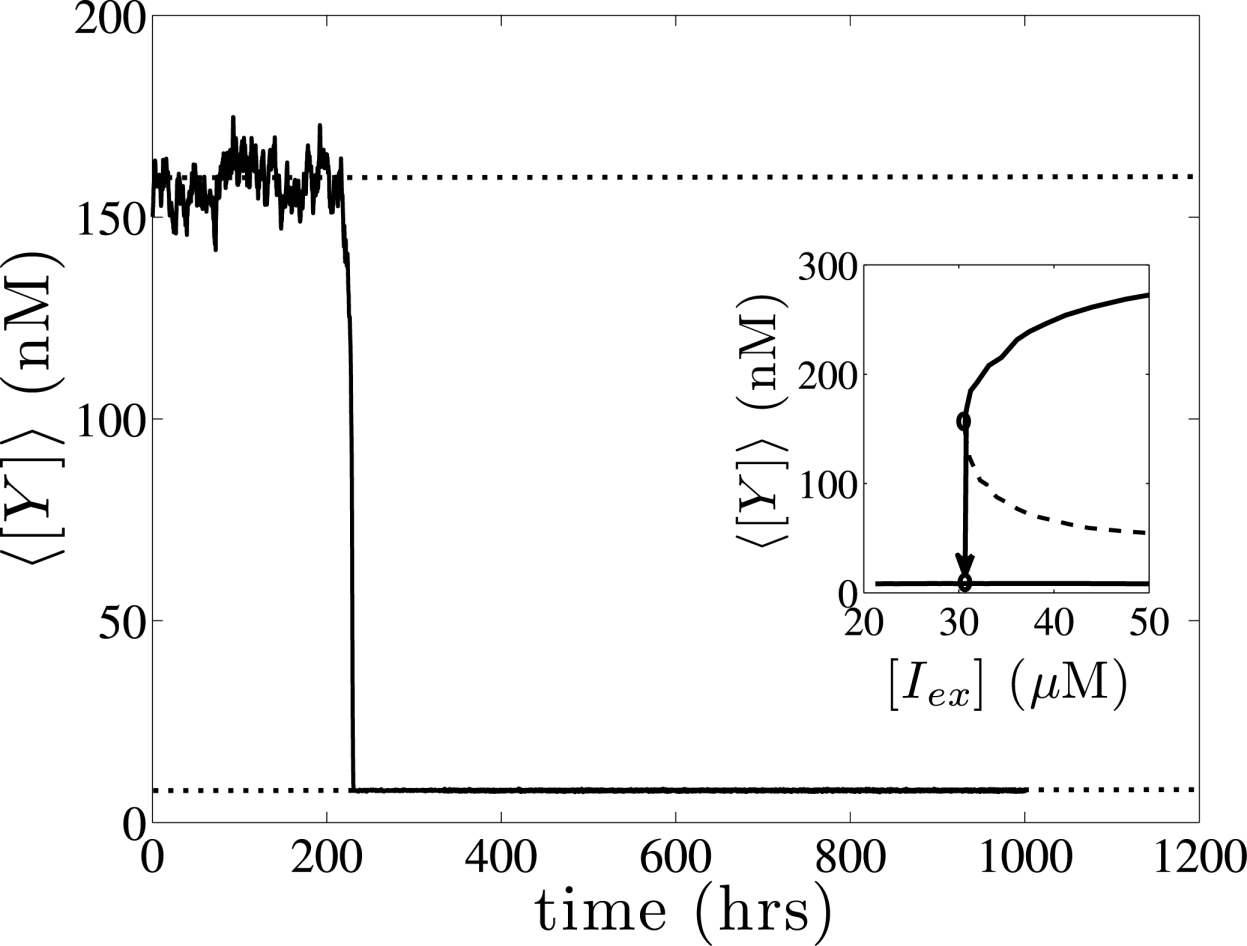
Noise induced phenotypic switching. A single stochastic simulation starting from a coarse steady state at [*I*_*ex*_] = 30.8*μ*M with ⟨[*Y*]⟩ = 157.2nM (upper branch). After the elapse of *a long time interval* (*t* ≈ 220hrs), stochastic noise induces a phenotypic switching towards an average value of ⟨[*Y*]⟩ = 7.9nM (lower branch). The inner figure shows the corresponding bifurcation diagram of the steady state average expression level of *lacY* as a function of [*I*_*ex*_], with the open circles representing the two different co-existing steady state solutions at [*I*_*ex*_] = 30.8*μ*M. Parameter set values: *m* = 3, *f* = 0.5 *π* = 0.03, *κ* = 0.05, *K* = 500 and y* = 50. CNMC simulations are performed with *N* = 10,000 cells.

As reported above, noise can induce rapid changes of the average phenotype of large cell populations, when the extracellular conditions are at the vicinity of critical turning point values. In Fig 12, we present a single copy simulation of a population at [*I*_*ex*_] = 30.8*μ*M, which initially fluctuates around an average phenotype of ⟨[*Y*]⟩ = 157.2nM (upper stable solution branch), for a long time period (*t* ≈ 220hrs) and then switches towards a lower expression level of *lacY* (⟨[*Y*]⟩ = 7.9nM, lower stable branch).

**Fig 13:**
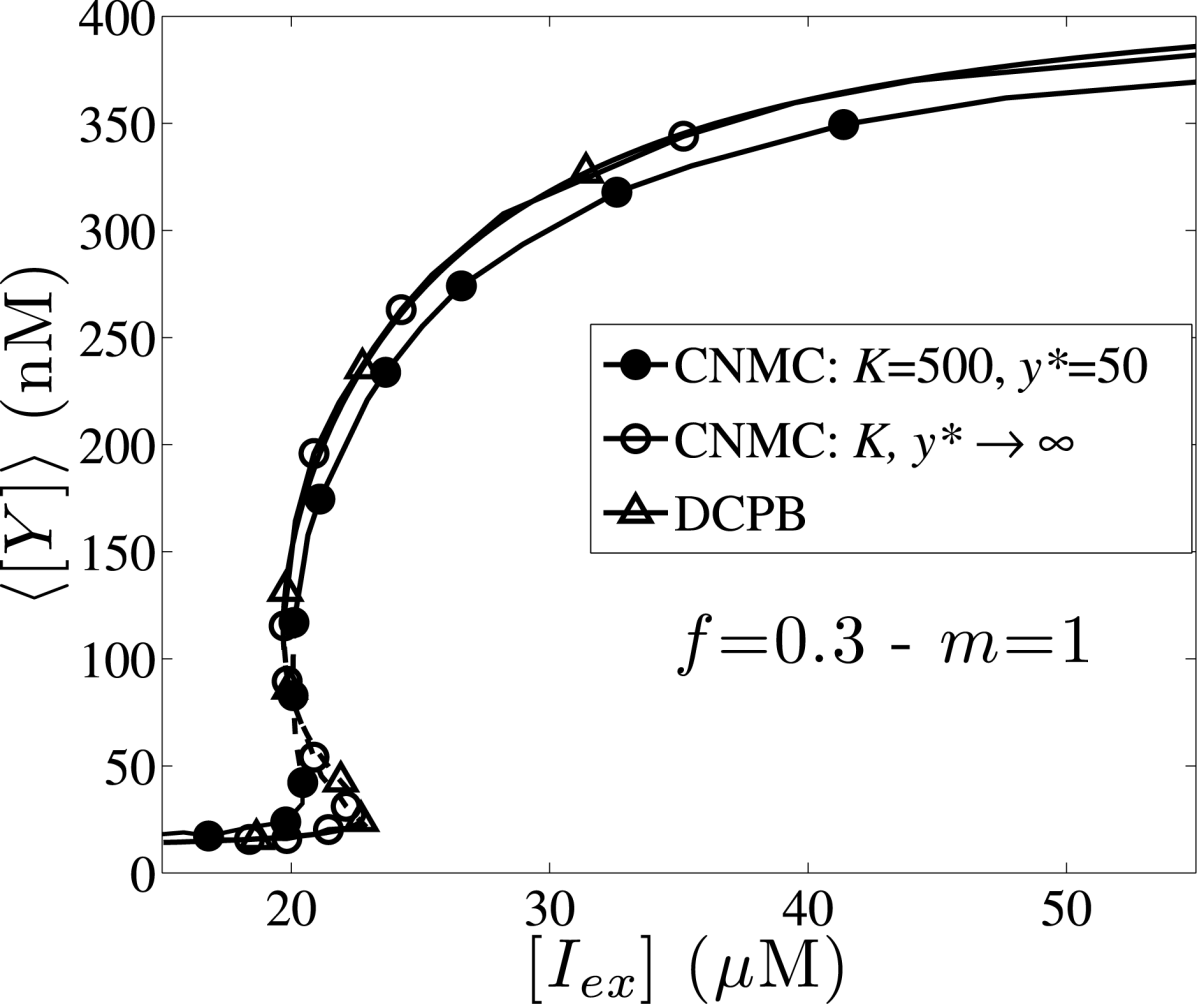
Effect of intrinsic noise for low single cell division rates. Steady state of average expression level, ⟨[*Y*]⟩, as a function of the external IPTG concentration for the case of *f* = 0.3 and *m* = 1. The *lines with full circles* correspond to the CNMC model with *K* = 500 and y* = 50; the *lines with open circles* correspond to the CNMC model neglecting intrinsic noise effects, and the *black lines* to the DCPB model. Parameter set values: *π* = 0.03 and *κ* = 0.05. CNMC simulations are carried out with *N* = 10,000 cells.

In all cases presented above, the bistability region is shifted towards higher [*I*_*ex*_] values by intensifying the effect of intrinsic noise. If we consider slower dynamics for the single cell division rate, then we observe a reverse effect. In Fig 13, we present the results obtained from the comparison of the CNMC model with *K* = 500, y* = 50, the CNMC neglecting intrinsic noise effects, and the DCPB model for the case of asymmetric partitioning, *f* = 0.3 and division rate *m* = 1. The DCPB and the CNMC model neglecting intrinsic heterogeneity show good agreement. When intrinsic noise effects are incorporated in the CNMC model, then the bistability region practically vanishes ([*I*_*ex*_] ∈ [20.45,20.5]*μ*M), with the upper turning point located at lower IPTG values (leftward shifting). When the single-cell division rate is relatively slow (*m* = 1) a low to high *lacY* expression level transition is expected to occur at a lower IPTG concentration value, when intrinsic noise effects are taken into consideration, whereas for higher cell-division rates intrinsic noise has always an opposite effect (low to high transitions occur at larger IPTG concentration values).

## Discussion

We present a multiscale computational methodology, which enables the systems-level study of cell populations simulated by means of stochastic models. In particular, a CNMC model is employed to simulate the dynamics of heterogeneous cell populations which carry the *lac* operon genetic network, featuring solution multiplicity over a range of extracellular inducer concentration values. In this work, we decompose the effect of different sources of heterogeneity (extrinsic and intrinsic) and study their effect on the bistability range of IPTG values. The extrinsic source of heterogeneity has been shown to have a significant effect on the phenotype of cell populations when performing deterministic cell population balance model computations [41]. However, the effect of intrinsic heterogeneity cannot be described by deterministic modelling since it involves the computation of Langevin stochastic differential equations for the intracellular reaction network. On the other hand, stochastic simulations which incorporate intrinsic noise effects cannot be used for a systems-level study of the problem; the number of stochastic simulations, which are required to compute accurately the range of bistability can be massive, rendering this approach as a computationally infeasible one.

To bypass this impediment, we employ the equation-free methodology, which utilises fine-scale information and projects it to a coarse—macroscopic level, for which well established numerical algorithms can be applied. Here, we perform bifurcation analysis, using pseudo arc-length continuation techniques in order to explore the dependence of the solution space on the extracellular inducer (IPTG) concentration, and accurately determine the range of bistability. Our stochastic CNMC-based computations are validated against deterministic descriptions, using large populations of cells (*N* = 10,000) and by neglecting the intrinsic noise effects (*K*, y* → ∞). Then, we explore the effect of intrinsic source of heterogeneity and demonstrate that when strengthened the range of bistability shrinks and shifts towards higher IPTG concentration values for sufficiently high single-cell division rates. When the effect of intrinsic noise is sufficiently strengthened, the turning point signalling the transition from low to high *lacY* expression levels *tends to infinity*, suggesting that the transition between high and low expression levels is irreversible, through modification of IPTG concentration values. A similar trend is also observed for populations dividing in a more asymmetric fashion. In addition, we show that the existence of intrinsic heterogeneity can lead to non-trivial dynamical behaviours such as phenotypic switching between co-existing stable steady states. We present such a transition at the neighbourhood of the bistability upper limit, when a sharp division rate is applied. Finally, we demonstrate the disappearance of the bistability interval for populations with low single-cell division rates and asymmetric partitioning mechanism, when intrinsic heterogeneity is also incorporated. In this case, the transition from low to high *lacY* expression levels occurs at a lower IPTG value compared to the case, where the intrinsic source of heterogeneity is neglected.

We also report that the equation-free framework is quite flexible to adopt for the performance of coarse-grained analysis of cell populations with genetic networks of increased complexity and different architectures. Indicatively, we report the cases of the *lac operon* genetic network with a promoter containing three repressor binding sites with cooperative interaction among them [64] and the genetic toggle switch [65]. The study of these networks requires new formulations for the single-cell reaction rate, the division rate and the partitioning mechanism; however, the application of the multiscale equation-free methodology does not require any modification and can be readily applied for the performance of their efficient systems level study.

## References

[1] Delbrück M. The burst size distribution in the growth of bacterial viruses (bacteriophages). J Bacteriol. 1945; 50: 131–135. 10.1128/JB.50.2.131-135.1945 20989330

[2] Chung JD, Stephanopoulos G. Studies of transcriptional state heterogeneity in sporulating cultures of *Bacillus subtilis* . Biotechnol Bioeng. 1995; 47: 234–242. 10.1002/bit.260470215 18623397

[3] Ptashne M. A genetic switch: gene control and phage lamda. Cambridge: Blackwell; 1987.

[4] Baek K, Svenningsen S, Eisen H, Sneppen K, Brown S. Single-cell analysis of *λ* immunity regulation. J Mol Biol. 2003; 334: 363–372. 10.1016/j.jmb.2003.09.037 14623180

[5] Warren L, Bryder D, Weissman IL, Quake SR. Transcription factor profiling in individual hematopoietic progenitors by digital RT-PCR. Proc Natl Acad Sci U S A. 2006; 103: 17807–17812. 10.1073/pnas.0608512103 17098862 PMC1693828

[6] Tischler J, Surani MA. Investigating transcriptional states at single-cell-resolution. Curr Opin Biotechnol. 2012; 24: 69–78. 10.1016/j.copbio.2012.09.013 23084076

[7] Rubakhin SS, Lanni EJ, Sweedler JV. Progress towards single cell metabolomics. Curr Opin Biotechnol. 2013; 24: 95–104. 10.1016/j.copbio.2012.10.021 23246232 PMC3545069

[8] Stewart GR, Robertson BD, Young DB. Tuberculosis: A problem with persistence. Nat Rev Microbiol. 2003; 1: 97–105. 10.1038/nrmicro749 15035039

[9] Balaban NQ, Merrin J, Chait R, Kowalik L, Leibler S. Bacterial persistence as a phenotypic switch. Science. 2004; 305: 1622–1625. 10.1126/science.1099390 15308767

[10] Weinberger LS, Burnett JC, Toettcher JE, Arkin AP, Schaffer DV. Stochastic gene expression in a lentiviral positive-feedback loop: HIV-1 Tat fluctuations drive phenotypic diversity. Cell. 2005; 122: 169–182. 10.1016/j.cell.2005.06.006 16051143

[11] Weinberger LS, Schenk T. An HIV feedback resistor: Auto-regulatory circuit deactivator and noise buffer. PLoS Biol. 2007; 5: e9 10.1371/journal.pbio.0050009 17194214 PMC1717016

[12] Weinberger LS, Dar RD, Simpson ML. Transient-mediated fate determination in a transcriptional circuit of HIV. Nat Genet. 2008; 40: 466–470. 10.1038/ng.116 18344999

[13] Collins TJ, Berridge MJ, Lipp P, Bootman MD. Mitochondria are morphologically and functionally heterogeneous within cells. EMBO J. 2002; 21: 1616–1627. 10.1093/emboj/21.7.1616 11927546 PMC125942

[14] das Neves RP, Jones NS, Andreu L, Gupta R, Enver T, Iborra FJ. Connecting variability in global transcription rate of mitochondrial variability. PLOS Biol. 2010; 8: e1000560 10.1371/journal.pbio.1000560 21179497 PMC3001896

[15] Johnston IG, Gaal B, das Neves RP, Enver T, Iborra FJ, Jones NS. Mitochondrial variability as a source of extrinsic cellular noise. PLoS Comput Biol. 2012; 8: e1002416 10.1371/journal.pcbi.1002416 22412363 PMC3297557

[16] Nevozhay D, Adams RM, Murphy KF, Josić K, Balázsi G. Negative autoregulation linearizes the dose-response and suppresses the heterogeneity of gene expression. Proc Natl Acad Sci U S A. 2009; 106: 5123–5128. 10.1073/pnas.0809901106 19279212 PMC2654390

[17] Toni T, Tidor B. Combined model of intrinsic and extrinsic variability for computational network design with application to synthetic biology. PLoS Comput Biol. 2013; 9: e1002960 10.1371/journal.pcbi.1002960 23555205 PMC3610654

[18] Hallen M, Li B, Tanouchi Y, Tan C, West M, You L. Computation of steady-state probability distributions in stochastic models of cellular networks. PLoS Comput Biol. 2011; 7: e1002209 10.1371/journal.pcbi.1002209 22022252 PMC3192818

[19] Mantzaris NV. From single-cell genetic architecture to cell population dynamics: quantitatively decomposing the effects of different population heterogeneity sources for a genetic network with positive feedback architecture. Biophys J. 2007; 92: 4271–4288. 10.1529/biophysj.106.100271 17384073 PMC1877777

[20] Block DE, Eitzman PD, Wangensteen JD, Srienc F. Slit scanning of *Saccharomyces cerevisiae* cells: quantification of asymmetric cell division and cell cycle progression in asynchronous culture. Biotechnol Prog. 1990; 6: 504–512. 10.1021/bp00006a015 1366842

[21] Aviziotis IG, Kavousanakis ME, Bitsanis IA, Boudouvis AG. Coarse-grained analysis of stochastically simulated cell populations with a positive feedback genetic network architecture. J Math Biol. 2015; 70: 1457–1484. 10.1007/s00285-014-0799-2 24929336

[22] Alberts B, Bray D, Lewis J, Raff M, Roberts K, Watson JD. Molecular biology of the cell. 3rd ed New York: Garland publishing; 1994.

[23] Paulsson J. Summing up the noise. Nature. 2004; 427: 415–418. 10.1038/nature02257 14749823

[24] Bruggeman FJ, Blüthgen N, Westerhoff HV. Noise management by molecular networks. PLoS Comput Biol. 2009; 5: e1000506 10.1371/journal.pcbi.1000506 19763166 PMC2731877

[25] Shah BH, Borwanker JD, Ramkrishna D. Monte Carlo simulation of microbial population growth. Math Biosci. 1976; 31: 1–23. 10.1016/0025-5564(76)90037-7

[26] Hatzis C, Srienc F, Fredrickson AG. Multistaged corpuscular models of microbial growth: Monte Carlo simulations. Biosystems. 1995; 36: 19–35. 10.1016/0303-2647(95)01524-O 8527693

[27] Mantzaris NV. Stochastic and deterministic simulations of heterogeneous cell population dynamics. J Theor Biol. 2006; 241: 690–706. 10.1016/j.jtbi.2006.01.005 16487980

[28] Smith M, Matsoukas T. Constant-number Monte Carlo simulation of population balances. Chem Eng Sci. 1998; 53: 1777–1786. 10.1016/S0009-2509(98)00045-1

[29] Shu CC, Chatterjee A, Hu WS, Ramkrishna D. Modeling of gene regulatory processes by population-mediated signaling: new applications of population balances. Chem Eng Sci. 2012; 70: 188–199. 10.1016/j.ces.2011.07.062 22581980 PMC3347889

[30] Stamatakis M, Zygourakis K. A mathematical and computational approach for integrating the major sources of cell population heterogeneity. J Theor Biol. 2010; 266: 41–61. 10.1016/j.jtbi.2010.06.002 20685607 PMC3175140

[31] Stamatakis M, Zygourakis K. Deterministic and stochastic population-level simulations of an artificial *lac* operon genetic network. BMC Bioinformatics. 2011; 12: 301 10.1186/1471-2105-12-301 21791088 PMC3181209

[32] Shu CC, Chatterjee A, Hu WS, Ramkrishna D. Role of intracellular stochasticity in biofilm growth. Insights from population balance modeling. PLoS One. 2013; 8: e79196 10.1371/journal.pone.0079196 24232571 PMC3827321

[33] Zechner C, Ruess J, Krenn P, Pelet S, Peter M, Lygeros J, et al Moment-based inference predicts bimodality in transient gene expression. Proc Natl Acad Sci U S A. 2012; 109: 8340–8345. 10.1073/pnas.1200161109 22566653 PMC3361437

[34] Zhang L, Strouthos CG, Wang Z, Deisboeck TS. Simulating brain tumor heterogeneity with a multiscale agent-based model: linking molecular signatures, phenotypes and expansion rates. Math Comput Model. 2009; 49: 307–319. 10.1016/j.mcm.2008.05.011 20047002 PMC2653254

[35] Murphy JT, Walshe R. Modeling antibiotic resistance in bacterial colonies using agent-based approach In: Dubitzky W, Southgate J, and Fuß H, editors. Procceedings of understanding the dynamics of biological systems. New York: Springer; 2011.

[36] Gorochowski TE, Matyjaszkiewicz A, Todd T, Oak N, Kowalska K, Reid S, et al BSim: an agent-based tool for modeling bacterial populations in systems and synthetic biology. PLoS One. 2012; 7: e42790 10.1371/journal.pone.0042790 22936991 PMC3427305

[37] Hellweger FL, Fredrick ND, Berges JA. Age-correlated stress resistance improves fitness of yeast: support from agent-based simulations. BMC Syst Biol. 2014; 8: 18 10.1186/1752-0509-8-18 24529069 PMC3927587

[38] Beckwith JR, Zipser D. The lactose operon. New York: Cold Spring Harbor Laboratory; 1970.

[39] Miller JH, Reznikoff WS. The operon. New York: Cold Spring Harbor Laboratory; 1978.

[40] Mantzaris NV. A cell population balance model describing positive feedback loop expression dynamics. Comput Chem Eng. 2005; 29: 897–909. 10.1016/j.compchemeng.2004.09.012

[41] Kavousanakis ME, Mantzaris NV, Boudouvis AG. A novel free boundary algorithm for the solution of cell population balance model. Chem Eng Sci. 2009; 64: 4247–4261. 10.1016/j.ces.2009.06.054

[42] Gear CW, Kevrekidis IG, Theodoropoulos C. Coarse integration/bifurcation analysis via microscopic simulators: micro-Galerkin methods. Comput Chem Eng. 2002; 26: 941–963. 10.1016/S0098-1354(02)00020-0

[43] Kevrekidis IG, Gear CW, Hyman JM, Kevrekidis PG, Runborg O, Theodoropoulos C. Equation-free, coarse-grained multiscale computation: enabling microscopic simulators to perform system-level analysis. Commun Math Sci. 2003; 1: 715–762. Available: http://projecteuclid.org/euclid.cms/1119655353 10.4310/CMS.2003.v1.n4.a5

[44] Kevrekidis IG, Gear CW, Hummer G. Equation-free: the computer-aided analysis of complex multiscale systems. AIChE J. 2004; 50: 1346–1355. 10.1002/aic.10106

[45] Keller HB. Numerical solution of bifurcation and nonlinear Eigenvalue problems In: Rabinowitz P, editor. Applications of Bifurcation Theory. New York: Academic Press; 1977.

[46] Kepler TB, Elston TC. Stochasticity in transcriptional regulation: origins, consequences and mathematical representations. Biophys J. 2001; 81: 3116–3136. 10.1016/S0006-3495(01)75949-8 11720979 PMC1301773

[47] Santillán M, Mackey MC. Influence of catabolite repression and inducer exclusion on the bistable behavior of the lac operon. Biophys J. 2004; 86: 1282–1292. 10.1016/S0006-3495(04)74202-2 14990461 PMC1303969

[48] Stamatakis M, Mantzaris NV. Comparison of deterministic and stochastic models of the lac operon genetic network. Biophys J. 2009; 96: 887–906. 10.1016/j.bpj.2008.10.028 19186128 PMC2996131

[49] Cohn M, Horibata K. Analysis of the differentiation and of the heterogeneity within a population of Escherichia coli undergoing induced *β*-galactosidase synthesis. J Bacteriol. 1959; 78: 613 13811042 10.1128/jb.78.5.613-623.1959 PMC290601

[50] Goeddel DV, Yansura DG, Caruthers MH. Binding of synthetic lactose operator DNAs to lactose repressors. Proc Natl Acad Sci U S A. 1977; 74: 3292–3296. 10.1073/pnas.74.8.3292 333432 PMC431535

[51] Elgart V, Kamenev A. Rare event statistics in reaction-diffusion systems. Phys Rev E. 2004; 70: 041106 10.1103/PhysRevE.70.041106 15600396

[52] Roma DM, O’Flanagan RA, Ruckenstein AE, Sengupta AM, Mukhopadhyay R. Optimal path to epigenetic switching. Phys Rev E. 2005; 71: 011902 10.1103/PhysRevE.71.011902 15697625

[53] Gillespie DT. A general method for numerically simulating the stochastic time evolution of coupled chemical reactions. J Comput Phys. 1976; 22: 403–434. 10.1016/0021-9991(76)90041-3

[54] Gillespie DT. Exact stochastic simulation of coupled chemical reactions. J Phys Chem. 1977; 81: 2340–2361. 10.1021/j100540a008

[55] Ramkrishna D. Population balances: Theory and applications to particulate systems in engineering. San Diego: Academic Press; 2000.

[56] Dien BS. Aspects of cell division cycle related behaviour of *Saccharomyces cerevisiae*. Growing in batch and continuous culture: A single-cell growth analysis. PhD Thesis, University of Minnesota, Minneapolis-St.Paul, MN. 1994.

[57] van Kampen NG. Itô versus Stratonovich. J Stat Phys. 1981; 24: 175–187. 10.1007/BF01007642

[58] Higham DJ. An algorithmic introduction to numerical simulation of stochastic differential equations. SIAM Rev. 2001; 43: 525–546. 10.1137/S0036144500378302

[59] Siettos CI, Armaou A, Makeev AG, Kevrekidis IG. Microscopic/stochastic timesteppers and coarse control: a kinetic Monte Carlo example. AIChE J. 2003; 49: 1922–1926. 10.1002/aic.690490727

[60] Gear CW. Projective integration methods for distributions. NEC technical report. Princeton, NJ. 2001; 2001–130. Available: http://www.princeton.edu/wgear/pdf.pdf

[61] Zou Y, Kavousanakis ME, Kevrekidis IG, Fox RO. Coarse-grained computation for particle coagulation and sintering processes by linking Quadrature Method of Moments with Monte-Carlo. J Comput Phys. 2010; 229: 5299–5314. 10.1016/j.jcp.2010.03.007

[62] Maeda YT, Masaki S. Regulatory dynamics of synthetic gene networks with positive feedback. J Mol Biol. 2006; 359: 1107–1124. 10.1016/j.jmb.2006.03.064 16701695

[63] Matsumoto Y, Ito Y, Tsuru S, Ying BW, Yomo T. Bacterial cells carrying synthetic dual-function operon survived starvation. Biomed Res Int. 2011; 2011: 489265–75. 10.1155/2011/489265 PMC3228684 22190854

[64] Santillán M. Bistable behaviour in a model of the *lac* operon in *Escherichia coli* with variable growth rate. Biophys J. 2008; 94: 2065–2081. 10.1529/biophysj.107.118026 18065471 PMC2257910

[65] Gardner TS, Cantor CR, Collins JJ. Construction of a genetic toggle switch in *Escherichia coli* . Nature. 2000; 403: 339–342. 10.1038/35002131 10659857

